# Cryo-EM structure of constitutively active human Frizzled 7 in complex with heterotrimeric G_s_

**DOI:** 10.1038/s41422-021-00525-6

**Published:** 2021-07-08

**Authors:** Lu Xu, Bo Chen, Hannes Schihada, Shane C. Wright, Ainoleena Turku, Yiran Wu, Gye-Won Han, Maria Kowalski-Jahn, Pawel Kozielewicz, Carl-Fredrik Bowin, Xianjun Zhang, Chao Li, Michel Bouvier, Gunnar Schulte, Fei Xu

**Affiliations:** 1 iHuman Institute, ShanghaiTech University, Shanghai, China; 2 School of Life Science and Technology, ShanghaiTech University, Shanghai, China; 3 Center for Excellence in Molecular Cell Science, Shanghai Institutes for Biological Sciences, Chinese Academy of Sciences, Shanghai, China; 4 University of Chinese Academy of Sciences, Beijing, China; 5 Section of Receptor Biology & Signaling, Department of Physiology & Pharmacology, Karolinska Institutet, Stockholm, Sweden; 6 Institute for Research in Immunology and Cancer, Department of Biochemistry and Molecular Medicine, Université de Montréal, Montréal, QC Canada; 7 Departments of Biological Sciences and Chemistry, Bridge Institute, University of Southern California, Los Angeles, CA USA; 8 Present Address: Orion Pharma R&D, Espoo, Finland

**Keywords:** Cryoelectron microscopy, Oncogenes

Dear Editor,

The ten mammalian Frizzleds (FZD_1–10_) belong to the class F of G protein-coupled receptors (GPCRs) and mediate WNT signaling through interaction with transducer proteins including Dishevelled (DVL) or heterotrimeric G proteins.^1^ Their involvement in human disease has put FZDs at the forefront of drug targets, especially anti-cancer therapy.^2^ However, no drugs have been developed for efficient pharmacological modulation of FZDs, partially owing to the limited understanding of FZD structure and activation mechanisms.^1,3^ Among class F, FZD_7_ is intensively pursued due to its relevance in various tumor models, particularly in intestinal cancers.^4^ Detailed structures of the receptor complexes would allow for structure-guided discovery of new drug candidates. FZD_1–10_ share structural similarity with the related class F member Smoothened (SMO), which mediates Hedgehog signaling and is a validated target for cancer therapy.^2^ In an effort to understand the structural basis of FZD activation and transducer interaction, we solved the structure of human FZD_7_ in complex with heterotrimeric mini G_s_ (mG_s_).^5^

Based on the evidence that FZD_7_ interacts with G_s_ to mediate muscle hypertrophy,^6,7^ we assessed its ability to activate heterotrimeric G_s_ independently of WNT stimulation. Co-expression of FZD_7_ with a bioluminescence resonance energy transfer (BRET)-based G_s_ biosensor,^8^ reporting the rearrangement or dissociation of Gα_s_ and Gβγ following receptor engagement and G protein activation, revealed that FZD_7_ exhibits constitutive activity similar to the class A β_2_-adrenoceptor (Fig. 1a; Supplementary information, Fig. S1a, b). Using an analogous assay that measures activity-dependent Gα_s_ translocation (Supplementary information, Fig. S1c), we found that the constitutive activity of FZD_7_ correlates with increased receptor expression (Supplementary information, Fig. S1d, e). Given the robust constitutive activity of FZD_7_ towards G_s_, we reconstituted purified, full-length human FZD_7_, heterotrimeric mG_s_ and Nanobody35 (Nb35), which stabilizes the nucleotide-free Gα_s_ and Gβ subunits,^9^ in the absence of ligand and obtained pure complexes following size exclusion chromatography (Supplementary information, Fig. S2). The final complex was composed of FZD_7_, mGα_s_, Gβ, Gγ and Nb35, which could be clearly identified by 2D classification (Fig. 1b; Supplementary information, Fig. S2d). We used single-particle cryo-EM analysis to determine the 3D structure of this complex. After several rounds of classification and auto-refinement, the resolution of the final structure reached 3.2 Å allowing us to build an atomic model based on the density map (Fig. 1c; Supplementary information, Figs. S3–S5, Table S1).

**Fig. 1:**
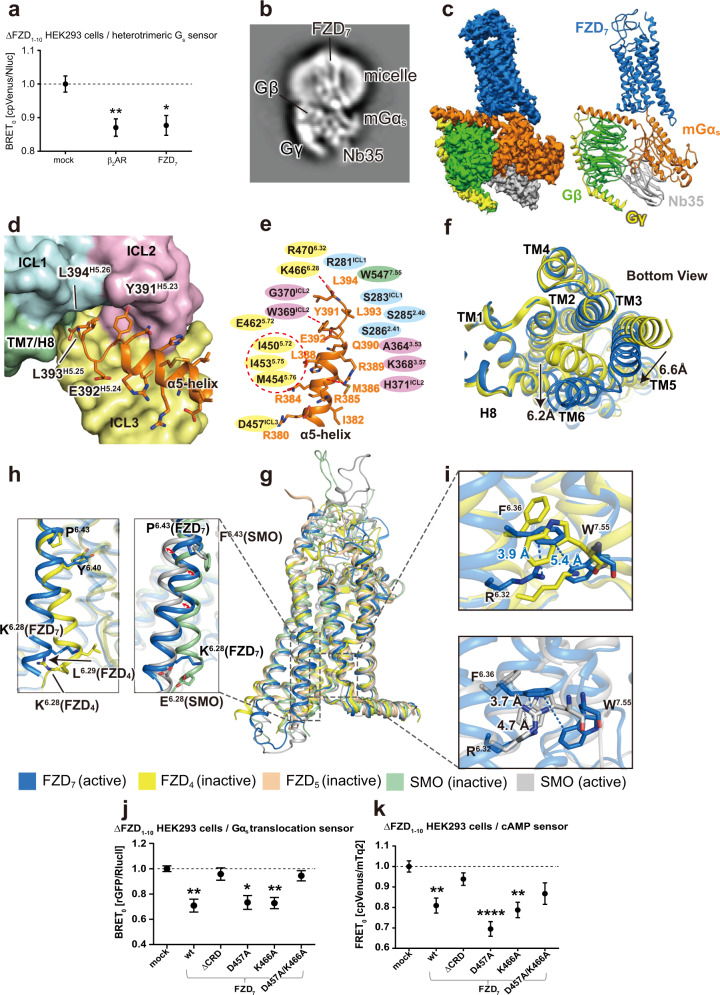
Structure of constitutively active FZD_7_ in complex with heterotrimeric mG_s_. **a** Normalized BRET_0_ values of ΔFZD_1–10_ HEK293 cells transiently co-transfected with the G_s_ BRET sensor along with either negative control (mock), the β_2_-adrenoceptor (β_2_AR) or FZD_7_. Data are represented as the means ± SEM of raw BRET_0_ that were obtained from simple linear regression of five independent experiments measured in quadruplicates shown in Supplementary information, Fig. S1A and normalized to the negative control. *P < 0.05; **P < 0.01 (one-way ANOVA followed by Sidak’s multiple comparison). **b** An annotated 2D class average of FZD_7_–mG_s_–Nb35 complex. **c** Overall density map and atomic model of FZD_7_–mG_s_–Nb35 complex (CRD was omitted due to linker flexibility). FZD_7_, blue; mGα_s_, orange; Gβ, green; Gγ, yellow; Nb35, gray. **d** Insertion of the α5-helix (mGα_s_, orange) into FZD_7_ helical bundle represented as surface (ICL1, blue; ICL2, pink; ICL3, yellow; TM7/H8, green). **e** Schematics of interactions between FZD_7_ and α5-helix. Hydrogen bonds are shown as red dashed lines. The red circle represents the hydrophobic interaction network. Yellow shades indicate residues that reside in TM5/6/ICL3; pink, TM3/4/ICL2; blue, TM1/2/ICL1; green, TM7/H8. **f** Superposition of FZD_7_ (blue) and FZD_4_ (yellow) structures, viewed from the intracellular side (bottom view). **g** Superposition of the active FZD_7_ structure (blue) with the inactive FZD_4_ (PDB: 6BD4, yellow), inactive FZD_5_ (PDB: 6WW2, light pink), active SMO (PDB: 6OT0, gray) and inactive SMO (PDB: 5V57, green) structures. **h** Comparison of the cytoplasmic portion of TM6 (from K^6.28^ to P^6.43^) in FZD_7_, FZD_4_, active SMO and inactive SMO structures. **i** R^6.32^, F^6.36^, W^7.55^ network in FZD_7_, FZD_4_ and active SMO structures. Blue dashed lines indicate the distance of F^6.36^–W^7.55^ and F^6.36^–R^6.32^ in FZD_7_. Gray dashed lines indicate the distance of R^6.32^–W^7.55^ and W^7.55^–F^6.36^ in active SMO structure. **j** Normalized BRET_0_ values of ΔFZD_1-10_ HEK293 cells transiently co-transfected with rGFP-CAAX and Gα_s_-67-*R*lucII, along with either negative control (mock), wild-type FZD_7,_ ΔCRD-FZD_7_ or the indicated FZD_7_ mutants. Data are represented as the means ± SEM of raw BRET_0_ that were obtained from simple linear regression of four independent experiments measured in quadruplicates shown in Supplementary information, Fig. S11b and normalized to the negative control. **P < 0.01; ***P < 0.001 (one-way ANOVA followed by Tukey’s multiple comparison). **k** Normalized FRET_0_ values of ΔFZD_1–10_ HEK293 cells transiently co-transfected with the FRET-based cAMP biosensor along with either negative control (mock), wild-type FZD_7,_ ΔCRD-FZD_7_ or the indicated FZD_7_ mutants. Data are represented as the means ± SEM of raw FRET_0_ that were obtained from simple linear regression of five independent experiments measured in quadruplicates shown in Supplementary information, Fig. S11c and normalized to the negative control. **P < 0.01; ****P < 0.0001 (one-way ANOVA followed by Sidak’s multiple comparison).

In accordance with the functional evidence for constitutive activity, the FZD_7_–mG_s_ complex structure provides the structural basis for ligand-independent G protein coupling (Fig. 1c). The interface between FZD_7_ and mG_s_ is dominated by the distal C-terminal segment of the α5-helix in mGα_s_ (Fig. 1d, e). The C-terminal leucine residues (L393^H5.25^, L394^H5.26^; superscripts refer to the residue position in the common Gα numbering scheme for G proteins/GPCRdb) are inserted into the helical bundle of the receptor. L393^H5.25^ and L394^H5.26^ establish extensive interactions with FZD_7_ residues yielding a locally converged network that stabilizes the complex (Fig. 1e). The terminal carboxyl group of L394^H5.26^ in mGα_s_ forms an ionic bond with K466^6.28^, and residues R281^ICL1^, K552^8.49^ and R470^6.32^ of FZD_7_ are located in close proximity (superscript numbers refer to the Ballesteros and Weinstein numbering system). Y391^H5.23^ forms a hydrogen bond with the backbone of W369^ICL2^. Residues I450^5.72^, I453^5.75^ and M454^5.76^ of FZD_7_ form a hydrophobic cleft accommodating L388^H5.20^. Furthermore, R385^H5.17^ forms an ionic bond with D457 in ICL3, further strengthening the interaction between the α5-helix and FZD_7_. In summary, the recognition of Gα_s_ by FZD_7_ is primarily governed by a network of hydrogen bonding and electrostatic interactions contributed from the C-terminal segment of the α5-helix (D381^H5.13^-L394^H5.26^), among which, interactions with L394^H5.26^ lock the α5-helix tail in an uncoiled, elongated conformation (Fig. 1e).

The placement of the α5-helix of mGα_s_ in the core of FZD_7_ stabilizes an open FZD_7_ conformation. We compared the FZD_7_–mG_s_ structure with the available inactive-state FZD_4_ crystal structure (PDB: 6BD4) and the inactive-state FZD_5_ cryo-EM structure (PDB: 6WW2) and observed a clear outward bending of TM6 and an inward shift of TM5 at the cytoplasmic side (Fig. 1f–h)—a conformational change characteristic of active-state class A and B GPCRs. This helical rearrangement is achieved through interaction of TM6 and TM5 with mG_s_ and opening of the molecular switch between TM6 and TM7 (R^6.32^/W^7.55^; Fig. 1i).^7^ Comparing inactive FZD_4_ with FZD_7_–mG_s_ reveals that the extracellular portion of TM6 of FZD_7_ extends above the surface of the lipid bilayer at an angle of 45° (Supplementary information, Figs. S6, S7), similar to what we have predicted in previous models^10^ and in contrast to the almost 90° bending in the FZD_4_ structure.^11^ Moreover, conserved cysteines within the hinge domain form disulfide bonds to both stabilize its structure and to link it with ECL1 (C210–C230; C234–C315^ECL1^) (Supplementary information, Fig. S6).^12^

To better understand the activation mechanism of FZD_7_ and G protein coupling to class F receptors, we compared the FZD_7_–mG_s_ structure with the agonist (24(*S*), 25-epoxycholesterol)-bound structure of SMO–G_i_^13^ (PDB: 6OT0). The helical arrangement at the upper portion of the FZD_7_ transmembrane core is more compact, presumably due to the absence of ligand (Supplementary information, Fig. S7). At the lower portion of TM6, substantially distinct conformations are observed between the SMO–G_i_ and FZD_7_–mG_s_ structures. Most strikingly, TM6 in SMO–G_i_ undergoes a parallel outward movement compared to inactive SMO, whereas TM6 in the FZD_7_–mG_s_ complex accomplishes a similar displacement of the cytoplasmic portion through a kink in the helix (Fig. 1h). The ionic interactions between TM6, ICL3 and the α5-helix of mGα_s_ (K466^6.28^–L394^H5.26^ and D457–R385^H5.17^) are likely to be the main contributors in maintaining this kink. In addition, Y478^6.40^ forms π–π interaction with W354^3.43^ to further maintain the bent TM6 conformation (Supplementary information, Fig. S7).

While the most evident structural rearrangements relate to TM6, additional positional shifts of TM2, TM3, TM4 and TM5 in the FZD_7_–mG_s_ complex are observed when compared to the SMO–G_i_ complex. These four helices constitute a more compact bundle in the FZD_7_–mG_s_ structure, partially stabilized by a network of π interactions (Supplementary information, Fig. S7e). In a cooperative manner, these interactions promote the cytoplasmic portion of TM4 shifting inward by ~2 Å (comparing the Cα of L383^4.47^ in FZD_7_–mG_s_ with corresponding L362^4.47^ in SMO–G_i_ complex structures) (Supplementary information, Fig. S7f, black arrow).

A conserved molecular switch between TM6 and TM7 was previously identified for all class F GPCRs, maintaining the receptor in an inactive conformation (observed as a hydrogen-bonding distance between R^6.32^ and the backbone of W^7.55^) in all inactive class F receptor structures.^7^ The polar interactions between R^6.32^ and W^7.55^ are broken in active SMO–G_i_ and the FZD_7_–mG_s_ complexes, resulting in a 6.4 Å distance between R470^6.32^ and W547^7.55^ in the FZD_7_–mG_s_ complex (Fig. 1f, h).^7^

To explore the conformational dynamics around the open and active FZD_7_ structure, we performed molecular dynamics (MD) simulations of FZD_7_ in complex with mG_s_393^5^ (Supplementary information, Fig. S8). Monomeric mG_s_ facilitated MD simulations due to its small size while minimizing the effect on receptor dynamics. These MD simulations allowed us to monitor general receptor integrity and the status of the molecular switch by assessing the angle of the kinked TM6 and the distance between R470^6.32^ and W547^7.55^. The overall hallmark of FZD_7_ activation — the kink in TM6 — is maintained over the time course of the simulation (measured as an angle between the backbone nitrogen atoms of V485^6.47^, P481^6.43^ and E462^6.24^). P^6.43^ is fully conserved among the FZD paralogues, but not in SMO (F^6.43^) (Supplementary information, Fig. S9). Analogous to P^6.50^ and P^6.47^ in class A and B receptors, respectively (Supplementary information, Fig. S10), P481^6.43^ is likely to contribute to the observed outward movement of the lower part of TM6^14^ (Fig. 1f, h). In the MD trajectories, the conformational changes of TM6 are manifested by the disruption of the molecular switch and a rearrangement of an extended aromatic network stabilizing the active receptor conformation (Supplementary information, Fig. S8). R470^6.32^ and the backbone oxygen atom of W547^7.55^ remain at over 8 Å distance throughout the simulation, rendering hydrogen bonding impossible between these two residues. Instead, R470^6.32^ is frequently bound with the carboxyl terminus of L^H5.26^ of mGα_s_. Interestingly, R470^6.32^ remains within hydrogen-bonding distance to the carboxyl terminus of L^H5.26^ more often than K466^6.28^, indicating that these positively charged residues lock the carboxyl tail between them (Supplementary information, Fig. S8d). This could contribute to the observed non-helical conformation of the tail of the α5-helix.

To gather functional evidence for the FZD_7_–mG_s_ interface and its role in maintaining the constitutive activity of FZD_7_ towards G_s_, we employed a mutagenesis-based approach in combination with assessment of Gα_s_ translocation and cAMP production as functional readouts of G_s_-dependent signaling. We focused on D457 (in ICL3) and K466^6.28^, which interact with the α5-helix of mGα_s_. Mutating either D457 or K466^6.28^ to alanine alone did not affect the constitutive activity of FZD_7_ on G_s_ translocation or cAMP production (Fig. 1j, k; Supplementary information, Figs. S11, S12). However, the double mutant D457A/K466^6.28^A abrogated FZD_7_ constitutive activity towards G_s_, suggesting that these mutations collectively interfere with G protein coupling. In contrast, the double mutant did not affect the ability of FZD_7_ to mediate WNT-induced activation of the WNT/β-catenin pathway as assessed by the TOPFlash reporter assay (Supplementary information, Fig. S13), underlining the concept of conformational selection for DVL-dependent signaling over G protein coupling as has been suggested previously.^7,15^

Although the CRD could not be resolved in the present structure, we observed that removal of the CRD (ΔCRD-FZD_7_) resulted in the inability to reconstitute the receptor–mG_s_ complex in vitro to the same extent as that of full-length FZD_7_ (Supplementary information, Fig. S14). Thus, we surmised that the CRD is required for FZD_7_–mG_s_ complex stability and that removal of the CRD could decrease constitutive activity. Therefore, we assessed the ability of the ΔCRD-FZD_7_ construct to functionally couple to G_s_ by assessing Gα_s_ translocation and cAMP production (Fig. 1j, k). Removal of the CRD blunted the constitutive activity towards G_s_ signaling as evidenced by the lack of Gα_s_ translocation and cAMP production. These data underline the requirement for the CRD to maintain constitutive activity of FZD_7_ towards heterotrimeric G proteins through intramolecular allostery.

In conclusion, we report the cryo-EM structure of FZD_7_–mG_s_ demonstrating how constitutive activity feeds into downstream signaling via heterotrimeric G proteins. With respect to the overall diversity among GPCRs, FZD_7_ has evolved a unique way to maintain certain homologous movements consistent with class A and B GPCR activation, while adapting its class-specific architecture to mediate G protein activation. While the classical hallmarks of G protein engagement are present in our structure, several differences can be found at the interface between the receptor and the G protein suggesting that FZDs harbor their own selectivity determinants for heterotrimeric G proteins. In short, the present structure of constitutively active FZD_7_–mG_s_, alongside previously published inactive structures of FZD_4_ and FZD_5_, opens the door to more accurate modeling of other FZDs and a platform for in silico drug discovery, which will aid in the discovery of new treatments to help those afflicted with diseases of WNT-FZD signaling.

## Supplementary information

Supplementary information

## Data Availability

The cryo-EM 3D map of the FZD_7_–mGs–Nb35 complex has been deposited in EMDB database with accession code EMD-31340; the coordinates have been deposited in PDB database with accession code 7EVW. The MD simulation data is available at www.gpcrmd.org with ID 245.
