# Supplementary material for: Cryo-EM structure of constitutively active human Frizzled 7 in complex with heterotrimeric Gs
Source: Cell Res. 2021 Jul 8;31(12):1311–4. doi: 10.1038/s41422-021-00525-6 (PMC8648716; doi:10.1038/s41422-021-00525-6)
Supplement: Supplementary file 1 — Supplementary information [file 41422_2021_525_MOESM1_ESM.pdf]

**Supplementary information**

## Methods

### Frizzled 7 ($FZD_7$) and $mG\alpha_s$, $G\beta$ and $G\gamma$ constructs

The human *FZD7* gene (Uniprot ID: O75084) was subcloned into the expression vector pFastBac1 vector. For the $FZD_7$ construct, the first 37 residues including the signal peptide (aa 1-32) were removed. HA and Flag tags were inserted on the N-terminus of $FZD_7$ (aa 38-574) to improve protein yield and detection. 10× His tag and AviTag$^{TM}$ were cloned on the C-terminus of $FZD_7$. The miniGs399 ($mG\alpha_s$) plasmid has been described previously[1,2]. We cloned $G\beta_1$ and $G\gamma_2$ into the pFastBac Dual vector. The 8× His was fused to the C-terminus of $G\beta_1$ for purification and C68S mutation introduced into the sequence of $G\gamma_2$ to increase solubilization of the protein. The Nb35 plasmid has been described previously[1,3].

### Protein expression and purification

$FZD_7$ protein was expressed and purified as previously described[4,5]. We used the Bac-to-Bac baculovirus system (Invitrogen) in *Spodoptera frugiperda* (Sf9) cells for expression. These cells were infected with baculovirus at a density of 2 × $10^6$ cells per ml. Cells were grown at 27 °C and collected 48h after infection. Cell membranes were washed with a low-salt buffer (10 mM HEPES (pH 7.5), 20 mM KCl, 10 mM $MgCl_2$ and protease inhibitor cocktail (Roche)) followed by three washes with a high-salt buffer (10 mM HEPES, pH 7.5, 1 M NaCl, 20 mM KCl, 10 mM $MgCl_2$ and protease inhibitor cocktail (Roche)). Next, the membranes were resuspended with a douncer after the addition of 2 mg/mL iodoacetamide (Sigma) at 4 °C for 1 h. After incubation, the solubilization buffer (100 mM HEPES (pH 7.5), 400 mM NaCl, 2% (w/v) *n*-docecyl-β-D-maltopyranoside (DDM) (Anatrace) and 0.2% (w/v) cholesteryl hemisuccinate (CHS) (Sigma)) was added to the membrane solution at 4 °C for 3 h. After centrifugation, the supernatant was incubated with TALON IMAC resin (Clontech) at 4 °C overnight. Then the resin was washed with 10 column volumes (CV) of buffer I: 50 mM HEPES (pH 7.5), 800 mM NaCl, 10% (v/v) glycerol, 0.5% (w/v) lauryl maltose neopentyl glycol (LMNG; Anatrace), 0.1% (w/v) CHS, 10 mM $MgCl_2$, 20 mM imidazole. The resin was then incubated with 2CV of Buffer I at 4 °C for 2 h to exchange DDM for LMNG. After incubation, the resin was washed with 10CV of buffer Ⅱ: 25mM HEPES (pH 7.5), 500 mM NaCl, 10% (v/v) glycerol, 0.03% (w/v) LMNG, 0.006% (w/v) CHS, 40 mM

imidazole. Finally, the resin was eluted with 4 CV of buffer III: 25 mM HEPES (pH 7.5), 300 mM NaCl, 10% (v/v) glycerol, 0.001% (w/v) LMNG, 0.0002% (w/v) CHS, 220 mM imidazole. Eluates were concentrated to 1 mg/mL and flash frozen in liquid nitrogen and stored at -80 °C for further use.

The $mG_s$ used in this project was the same as that used in the GPR52-$mG_s$-Nb35 and $A_2AR$–$mG_s$-Nb35 structures. In brief, $mG\alpha_s$ protein was expressed in the *E. coli* strain BL21. Cells were collected by centrifugation at 3500 rpm for 20 min and lysed by sonication. After another centrifugation, the supernatant was purified by $Ni^{2+}$ affinity chromatography, followed by cleavage of the His-tag using TEV protease and reverse purification on $Ni^{2+}$-NTA agarose to remove the TEV and undigested $mG\alpha_s$. Finally, the sample was loaded into a HiLoad16/60 column to obtain purified $mG\alpha_s$ protein. The purified protein was subsequently concentrated to 3 mg/mL and flash frozen in liquid nitrogen and stored at -80 °C for further use. The Gβγ and Nb35 purification was performed following previously described protocols[2,3] .

Purified $FZD_7$ protein was mixed with $mG\alpha_s$, Gβγ, Nb35 at a 1:1.3:1.5:2 molar ratio. This mixture was incubated at 4 °C overnight, followed by the addition of apyrase to catalyze the hydrolysis of unbound GDP to stabilize the nucleotide-free complex. To remove excess $mG\alpha_s$, Gβγ and Nb35 protein, the mixture was purified by size-exclusion chromatography with buffer containing 20 mM HEPES (pH 7.5), 100 mM NaCl, 0.00075% (w/v) LMNG, 0.00025% (w/v) CHS and 0.00025% (w/v) GDN. Peak fractions were concentrated to ~1-2 mg/mL for electron microscopy analysis.

**Cryo-EM sample preparation**

The freshly purified protein sample was added to a glow-discharged holy carbon grid (Quantifoil, 400 mesh gold R1.2/1.3). After adding the sample, the grid was blotted 4s with filter paper. Then the grid was immersed in liquid ethane with a FEI Vitrobot Mark IV at 100% humidity, 8 °C.

**Data collection**

All the datasets were collected on a Titan Krios 300 kV electron microscope (Thermo Fisher Scientifics, USA) equipped with a GIF Quantum energy filter (20 eV energy slit width, Gatan Inc., USA) and a K2 Summit direct electron detector (Gatan Inc, USA). For the $FZD_7$-$mG_s$-Nb35 sample,

a total of 5872 movies were recorded at 130k nominal magnification (calibrated pixel size: 1.04 Å/pixel) and super resolution mode by SerialEM using the beam image shift acquisition method with one image near the edge of each hole and saved as non-gain normalized TIFF files. A 50-µm C2 aperture was always inserted during the data collection period. The defocus ranged from -0.7 to -2.2 µm. For each movie stack, a total of 40 frames were recorded at a dose rate of 8 $e^-$/pixel/sec for a duration of 8.1s, yielding a total dose of 60 $e^-/A^2$.

**Data processing**

Both datasets were motion corrected with MotionCor2[6] using 5 × 5 patches and no frame grouping. Both the dose weighted and non-dose weighted averages were saved, and the CTF parameters were estimated based on the non-dose-weighted averages using CTFFIND4[6,7]. Only images with the highest resolution less than 4 Å were selected for further processing. Moreover, images with visible contamination or large carbon regions by manual examination were also removed. For the $FZD_7$-$mG_s$-Nb35 dataset, a total of 4462 movies were finally chosen for particle picking. To avoid potential bias about the structural conformation in the dataset, a Laplacian-of-Gaussian blob picker was firstly applied to pick particles. Good 2D class averages with randomized orientations and clear secondary features were selected as the 2D templates for another round of auto-picking process, yielding an initial particle stack of 2,074,920 particles. Another round of 2D classification was applied to eliminate bad quality particles by Relion 3.1[8], yielding a dataset containing 700,220 good quality particles with randomized orientations and clear secondary features. These particles were imported into cryoSPARC 2.15[9] to generate five *de novo* initial models, and one model out of five from which 2D projection matches the majority of 2D class averages was selected as the initial model for further processing by Relion3.1[8]. For the first round of 3D classification, this initial model was firstly low-pass filtered by 20 Å and used to divide the dataset into 3 different 3D classes. To overcome problems with conformational heterogeneity, the particles with reasonable 3D reconstructed maps were then grouped before subsequent rounds of 3D classifications focusing on the transmembrane domains. The centering, clipping, binning, thresholding operations of the maps; and the generation, addition, multiplication of masks were performed by EMAN2[10]. Homemade python (https://www.python.org/) scripting was also applied to eliminate particles that could not converge to the correct Euler angles. Finally, a total of 307,000 particles were selected for homogeneous refinement and post-processing, yielding a map with

resolution of 3.3 Å at a Fourier shell correlation of 0.143. Then the dataset was subjected to further CTF refinement and post-processing, and the final resolution was improved to 3.2Å. Subsequently, classification without alignment focusing on the transmembrane region was implemented to further separate particles into 3 different groups. 224,750 particles which have high resolution features at the transmembrane region were selected and were subject to final rounds of homogeneous refinement. The local resolution was estimated using the cryoSPARC v2.15[9] "local resolution estimation" function.

**Model Building**

The homology models of the human $FZD_7$, $mG\alpha_s$ were initially generated by Swiss model[11] (template PDB: 5V57[5], 7D3S[12]). For Gβ, Gγ and Nb35, the model 3CIK[13], 6PCV[14] and 6GDG[1] were chosen. These models were then fitted into the density maps in UCSF Chimera[15], and manually adjusted to fit the density maps in Coot software[16]. Subsequently, the generated model was automatically refined and manually adjusted in Coot[16] and Phenix[17], respectively, for several iterations. The clashscores, Molprobity and Ramachandran analysis was performed using the MolProbity[18]. The final refinement statistics were generated using the "comprehensive validation (cryo-EM)" function in Phenix[17]. To avoid potential overfitting of the model, both FSCs between the model vs the half1 map (work) and the model vs the half2 map (test) were determined (FSC=0.5 cutoff). Structural figures were prepared in Chimera[15], Coot[16] and Pymol (http://www.pymol.org).

The final model of $FZD_7$-$mG_s$ complex revealed seven intact transmembrane helices (7TM), extracellular loops 1 and 2 (ECL1 and 2), the entire hinge domain[19] (aa 210-250), the three intracellular loops (ICLs), the helix 8 perpendicular to the core helices, the $mG_s$ heterotrimer and Nb35. The extracellular N N-terminus including the CRD (aa 47-168) and ECL3 (aa 509-525) were not resolved, likely owing to structural flexibility relative to the receptor core.

**Molecular dynamics (MD) simulations**

To obtain a simulation complex of suitable size, the $mG\alpha_s$-Gβ-Gγ-Nb35 complex was replaced by $miniG_s393$ ($mG\alpha_s$)[19]. The $FZD_7$-$miniG_s393$ system was built using Schrödinger Maestro 2020-4 molecular modeling platform (Schrödinger, LLC, New York, NY, 2020). The unresolved ECL3 of $FZD_7$ was modelled using the SMO structure (PDB ID: 5L7D) as a template and $miniG_s393$ using

the original $mG_s$ as a template. For unresolved amino acids at $mG_s$ structure, mini$G_s$393 structure (PDB ID: 5G53) guided the model building.

The MD simulations were run using GROMACS 2020.4[20]. The receptor was oriented using the OPM database[21] and embedded in the POPC lipid bilayer (151 lipids / leaflet) by CHARMM-GUI server[22] with TIP3p water molecules and 0.15 M NaCl. The system was minimized for approximately 3000 steps and then equilibrated with gradually decreasing position restraints on protein and lipid components. In the last 50 ns of the equilibration run, the harmonic force constants of 50 kJ $mol^{-1}$ $nm^{-2}$ were applied on the protein atoms only. It should be noted that the simulations were run in absence of the Gβγ subunits and Nb35.

Ten independent isobaric and isothermic (NPT) ensemble production simulations of 50 ns each were initiated from random velocities using the CHARMM36m force field[23] and a 2-fs time step. The temperature at 303.15 K was maintained with Nose-Hoover thermostat[24] and the pressure at 1 bar with Parrinello-Rahman barostat[25]. Potential-shift-Verlet was used for electrostatic and van der Waals interactions with 12 Å cut-off, and the bonds between hydrogen and other atoms were constrained by the LINCS algorithm[26]. The data were analyzed using VMD (visualization and measurement of RMSDs, distances and angles)[27] and visualized in PyMol. MD simulations trajectories will be available at GPCRmd (an open-access MD database for GPCRs; www.gpcrmd.org).

**Plasmids and molecular cloning**

The FRET-based cAMP sensor (H187) was from Kees Jalink (The Netherlands Cancer Institute, Amsterdam, The Netherlands)[28]. HiBiT-$FZD_7$ and ΔCRD-HiBiT-$FZD_7$ were generated by replacing $FZD_4$ in pcDNA3.1 HiBiT-$FZD_4$[29] with $FZD_7$ or ΔCRD-$FZD_7$ from SNAP-$FZD_7$ (Heptares) by Gibson assembly. $FZD_7$ point mutants were cloned using the GeneArt Site-Directed Mutagenesis Kit (Thermo Fisher Scientific) with the following primers: HiBiT-$FZD_7$-D457A forward primer 5'-CCATCATGAAACACGCCGGCACCAAGACCGA-3', HiBiT-$FZD_7$-D457A reverse primer 5'-TCGGTCTTGGTGCCGGCGTGTTTCATGATGG-3', HiBiT-$FZD_7$-K466A forward primer 5'-ACCGAGAAGCTGGAGGCGCTCATGGTGCGCAT-3' and HiBiT-$FZD_7$-

K466A reverse primer 5'-ATGCGCACCATGAGCGCCTCCAGCTTCTCGGT-3'. All constructs were verified by sequencing (Eurofins genomics).

**Reagents**

NanoLuciferase substrate furimazine (# N1572) and Dual-Luciferase Reporter Assay System (# E1910) were purchased from Promega. Coelenterazine 400a (FC36331) was purchased from Biosynth. C59 (2-[4-(2-Methylpyridin-4-yl)phenyl]-N-[4-(pyridin-3-yl)phenyl]acetamide) porcupine inhibitor[30] was from Abcam (# ab142216). Lipofectamine 2000 transfection reagent, Phusion Polymerase and GeneArt™ site-directed mutagenesis kit (# A13312) were purchased from Thermo Fisher Scientific. Dulbecco's Modified Eagle's Medium (DMEM) was from Gibco. Opaque white, F-bottom 96-well plates and opaque black 96-well plates were from Greiner BioOne. $\Delta FZD_{1-10}$ HEK293 cells were kindly provided by Benoit Vanhollebeke (Université libre de Bruxelles, Brussels, Belgium)[31].

**Cell culture**

$\Delta FZD_{1-10}$ HEK293T (human embryonic kidney) cells[31] were used to functionally assess $FZD_7$ and associated mutants using a panel of biosensors. Cells were grown in Dulbecco's Modified Eagle's Medium (DMEM) supplemented with 2 mM glutamine, 10% fetal calf serum, 0.1 mg/mL streptomycin, and 100 units/mL penicillin at 37 °C with 5% $CO_2$.

**Transient transfection and plating**

Cells (300,000 cells/mL) were transfected in suspension with a total of 1 µg DNA/mL cell suspension using Lipofectamine 2000 (Thermo Fisher Scientific, Waltham, MA, USA). For experiments using the heterotrimeric $G_s$ or EPAC-based cAMP sensor[28], 500 ng negative control or GPCR was co-transfected along with 500 ng of the BRET-($G_s$) or FRET-based (cAMP) biosensor. For the $G\alpha_s$ translocation assay[32], cells were transfected with either ssDNA or receptor, $G\alpha_s$-67-*R*lucII and rGFP-CAAX in a (5:1:30) ratio. ssDNA was supplemented to yield 1 µg total DNA/mL cell suspension. Cells mixed with the transfection reagents were seeded onto PDL-coated 96-well plates and incubated at 37 °C with 5% $CO_2$. 24 hours prior to the experiment, C59 was added to a final concentration of 10 nM. Opaque white 96-well plates were used for all BRET HiBiT-$FZD_7$ surface expression experiments. Opaque black 96-well plates were used for

experiments with the FRET-based cAMP biosensor. The porcupine inhibitor C59 was added to prevent the secretion of endogenously produced WNTs in order to exclude autocrine and paracrine, ligand-dependent stimulation.

**$BRET_0$-based assessment of $G_s$ activity**

Transfected cells grown for 48 hours in 96-well plates were washed with HBSS and incubated with 1/1,000 dilution of furimazine stock solution. After incubation for 3 min, the BRET ratio was measured in two consecutive reads to assess constitutive receptor activity. All experiments were conducted at 37 °C with a CLARIOstar plate reader. Nluc emission intensity was selected using a 450/40 nm monochromator (Gain: 3600) and cpVenus[173] emission using a 535/30 nm monochromator (Gain: 4000) with an integration time of 0.3 seconds in both channels. All experiments were conducted in quadruplicates and BRET ratios were defined as acceptor emission/donor emission. Nluc/BRET plots were fitted using simple linear regression in Prism 5.0 software (GraphPad, San Diego, CA, USA) and $BRET_0$ was defined as the Y-intercept with its computed standard error resulting from the linear fit of BRET values over increasing Nluc intensities.

**$BRET_0$-based assessment of $G\alpha_s$ translocation**

Transfected cells grown for 48 hours in 96-well plates were washed with HBSS and incubated with 2.5 µM coelenterazine 400a. After incubation for 5 min, the BRET ratio was measured to assess constitutive receptor activity. All experiments were conducted at 37 °C with a Tecan Spark plate reader. *R*lucII emission intensity was selected using a 400/40 nm monochromator and rGFP emission using a 540/35 nm monochromator with an integration time of 50 ms in both channels. All experiments were conducted in quadruplicates and BRET ratios were defined as acceptor emission/donor emission. *R*lucII/BRET plots were fitted using a simple linear regression in Prism 5.0 software (GraphPad, San Diego, CA, USA) and $BRET_0$ was defined as the Y-intercept with its computed standard error resulting from the linear fit of BRET values over increasing *R*lucII intensities.

**$FRET_0$-based assessment of cAMP accumulation**

Transfected cells grown for 48 hours in 96-well plates were washed with HBSS and incubated with 100 μL HBSS. The FRET ratio was measured in two consecutive reads for the assessment of constitutive receptor activity. All experiments were conducted at 37 °C with a CLARIOstar plate reader. mTurquoise2 was excited at 430/20 nm and its emission intensity was selected using a 480/200 nm monochromator (Gain: 1300). cpVenus[173] emission was recorded using a 535/30 nm monochromator (Gain: 1200). 40 excitation flashes were employed per data point. All experiments were conducted in quadruplicates and FRET ratios were defined as acceptor emission/donor emission. mTurquoise2/FRET plots were fitted using simple linear regression in Prism 5.0 software (GraphPad, San Diego, CA, USA) and $FRET_0$ was defined as the Y-intercept with its computed standard error resulting from the linear fit of FRET values over increasing mTurquoise2 intensities.

**Luminescence-based assessment of receptor surface expression**

$\Delta FZD_{1-10}$ HEK 293T cells were transiently transfected in suspension using Lipofectamine 2000 (Thermo Fisher Scientific). $2 \times 10^5$ cells/mL were transfected with 500 ng of HiBiT-$FZD_7$ and 500 ng of pcDNA plasmid DNA. 100 μL of cells were seeded onto a PDL-coated opaque white 96-well plates (Greiner Bio-One). Forty-eight hours post-transfection, cells were washed once with HBSS (HyClone) after which 100 μL of HiBiT Lytic Buffer (Promega, N3040) containing 1:100 HiBiT Lytic Substrate (Promega, N3040) and 1:200 LgBiT Protein (Promega, N3040) was added and incubated for 10 min. Luminescence was then read at 475-30 nm with a CLARIOstar microplate reader (BMG LABTECH). All experiments were conducted in triplicates.

**Densitometric quantification of protein bands**

The complex sample fractions after size-exclusion chromatography were mixed with protein loading buffer and prepared for SDS-PAGE (Genscript). Proteins were visualized with Coomassie blue. The gels were scanned and exported as image files. The bands were quantified by densitometry using image J software (NIH) (www.imagej.net). Data are presented as means ± SEM. and *P*-values of < 0.05 were considered significant. Student's *t*-test was performed to compare the differences between two groups. Densitometry measurements of $mG\alpha_s/FZD_7$ bands are based on three independent SDS-PAGE results, and those of $mG\alpha_s/\Delta CRD\text{-}FZD_7$ bands are based on four independent SDS-PAGE results.

**TOPFlash reporter gene assay for assessment of WNT-induced β-catenin signaling**

$\Delta FZD_{1\text{-}10}$ HEK 293T cells were transiently transfected in suspension using Lipofectamine 2000 (Thermo Fisher Scientific). $2 \times 10^5$ cells/mL were transfected with 500 ng of HiBiT-$FZD_7$ wildtype or the D457A/K466 double mutant along with 400 ng M50 Super 8×TOPFlash, 100 ng pRL-TK Luc. 100 µL of cells were seeded onto a PDL-coated opaque white 96-well plates (Greiner Bio-One). Twenty-four hours after transfection, cells were washed with 100 µL HBSS and incubated for four hours in 72 µL/well of FBS-reduced (0.5 %) DMEM supplemented with 10 nM C59. Thereafter, 8 µL of increasing concentrations of recombinant WNT-3A (in 0.1 % BSA/HBSS) or respective vehicle control were added. Twenty-four hours after stimulation, cells were washed with HBSS and lysed in 30 µL of Promega's dual luciferase passive lysis buffer. Subsequently, 20 µL luciferase assay reagent (LARII) was added to each well and β-catenin-dependent *Firefly* luciferase (Fluc) intensity was measured using a CLARIOstar microplate reader (580/80 nm; 1 sec integration time). Next, 20 µL Stop&Glo Reagent was added to quantify *Renilla* luciferase (*R*luc) emission intensity (480/80 nm; 1 sec integration time) to control for variations in cell number and transfection efficiency.

**References**


1 Garcia-Nafria, J., Lee, Y., Bai, X., Carpenter, B. & Tate, C. G. Cryo-EM structure of the adenosine A2A receptor coupled to an engineered heterotrimeric G protein. *Elife* **7**, doi:10.7554/eLife.35946 (2018).

2 Lin, X. *et al.* Structural basis of ligand recognition and self-activation of orphan GPR52. *Nature* **579**, 152-157, doi:10.1038/s41586-020-2019-0 (2020).

3 Carpenter, B. & Tate, C. G. Engineering a minimal G protein to facilitate crystallisation of G protein-coupled receptors in their active conformation. *Protein Eng Des Sel* **29**, 583-594, doi:10.1093/protein/gzw049 (2016).

4 Lin, X. *et al.* Structural basis of ligand recognition and self-activation of orphan GPR52. *Nature* **579**, 152-+, doi:10.1038/s41586-020-2019-0 (2020).

5 Zhang, X. *et al.* Crystal structure of a multi-domain human smoothened receptor in complex with a super stabilizing ligand. *Nat Commun* **8**, 15383, doi:10.1038/ncomms15383 (2017).

6 Zheng, S. Q. *et al.* MotionCor2: anisotropic correction of beam-induced motion for improved cryo-electron microscopy. *Nat Methods* **14**, 331-332, doi:10.1038/nmeth.4193 (2017).

7 Rohou, A. & Grigorieff, N. CTFFIND4: Fast and accurate defocus estimation from electron micrographs. *Journal of structural biology* **192**, 216-221, doi:10.1016/j.jsb.2015.08.008 (2015).

8 Scheres, S. H. RELION: implementation of a Bayesian approach to cryo-EM structure determination. *Journal of structural biology* **180**, 519-530, doi:10.1016/j.jsb.2012.09.006 (2012).

9 Punjani, A., Rubinstein, J. L., Fleet, D. J. & Brubaker, M. A. cryoSPARC: algorithms for rapid unsupervised cryo-EM structure determination. *Nat Methods* **14**, 290-296, doi:10.1038/nmeth.4169 (2017).

10 Tang, G. *et al.* EMAN2: an extensible image processing suite for electron microscopy. *Journal of structural biology* **157**, 38-46, doi:10.1016/j.jsb.2006.05.009 (2007).

11 Guex, N., Peitsch, M. C. & Schwede, T. Automated comparative protein structure modeling with SWISS-MODEL and Swiss-PdbViewer: a historical perspective. *Electrophoresis* **30 Suppl 1**, S162-173, doi:10.1002/elps.200900140 (2009).

12 Fukuhara, S. *et al.* Structure of the human secretin receptor coupled to an engineered heterotrimeric G protein. *Biochemical and biophysical research communications* **533**, 861-866, doi:10.1016/j.bbrc.2020.08.042 (2020).

13 Tesmer, J. J., Tesmer, V. M., Lodowski, D. T., Steinhagen, H. & Huber, J. Structure of human G protein-coupled receptor kinase 2 in complex with the kinase inhibitor balanol. *Journal of medicinal chemistry* **53**, 1867-1870, doi:10.1021/jm9017515 (2010).

14 Cash, J. N. *et al.* Cryo-electron microscopy structure and analysis of the P-Rex1-Gbetagamma signaling scaffold. *Sci Adv* **5**, eaax8855, doi:10.1126/sciadv.aax8855 (2019).

15 Pettersen, E. F. *et al.* UCSF Chimera--a visualization system for exploratory research and analysis. *J Comput Chem* **25**, 1605-1612, doi:10.1002/jcc.20084 (2004).

16 Emsley, P., Lohkamp, B., Scott, W. G. & Cowtan, K. Features and development of Coot. *Acta Crystallogr D Biol Crystallogr* **66**, 486-501, doi:10.1107/S0907444910007493 (2010).

17 Adams, P. D. *et al.* PHENIX: a comprehensive Python-based system for macromolecular structure solution. *Acta Crystallogr D Biol Crystallogr* **66**, 213-221, doi:10.1107/S0907444909052925 (2010).

18 Chen, V. B. *et al.* MolProbity: all-atom structure validation for macromolecular crystallography. *Acta Crystallogr D Biol Crystallogr* **66**, 12-21, doi:10.1107/S0907444909042073 (2010).

19 Nehme, R. *et al.* Mini-G proteins: Novel tools for studying GPCRs in their active conformation. *PLoS One* **12**, e0175642, doi:10.1371/journal.pone.0175642 (2017).

20 Berendsen, H. J. C., Vanderspoel, D. & Vandrunen, R. Gromacs - a Message-Passing Parallel Molecular-Dynamics Implementation. *Computer Physics Communications* **91**, 43-56, doi:Doi 10.1016/0010-4655(95)00042-E (1995).

21 Lomize, M. A., Pogozheva, I. D., Joo, H., Mosberg, H. I. & Lomize, A. L. OPM database and PPM web server: resources for positioning of proteins in membranes. *Nucleic Acids Res* **40**, D370-376, doi:10.1093/nar/gkr703 (2012).

22 Jo, S., Lim, J. B., Klauda, J. B. & Im, W. CHARMM-GUI Membrane Builder for mixed bilayers and its application to yeast membranes. *Biophys J* **97**, 50-58, doi:10.1016/j.bpj.2009.04.013 (2009).

23 Huang, J. *et al.* CHARMM36m: an improved force field for folded and intrinsically disordered proteins. *Nat Methods* **14**, 71-73, doi:10.1038/nmeth.4067 (2017).

24 Nosé, S. & Klein, M. L. Constant pressure molecular dynamics for molecular systems. *Molecular Physics* **50**, 1055-1076 (1983).

25 Parrinello, M. & Rahman, A. Polymorphic transitions in single crystals: A new molecular dynamics method. *J. Appl. Phys.; (United States)*, Medium: X; Size: Pages: 7182-7190 (1981).

26 Hess, B., Bekker, H., Berendsen, H. J. & Fraaije, J. G. LINCS: a linear constraint solver for molecular simulations. *Journal of computational chemistry* **18**, 1463-1472 (1997).

27 Humphrey, W., Dalke, A. & Schulten, K. VMD: visual molecular dynamics. *J Mol Graph* **14**, 33-38, 27-38 (1996).

28 Klarenbeek, J., Goedhart, J., van Batenburg, A., Groenewald, D. & Jalink, K. Fourth-generation epac-based FRET sensors for cAMP feature exceptional brightness, photostability and dynamic range: characterization of dedicated sensors for FLIM, for ratiometry and with high affinity. *PLoS One* **10**, e0122513, doi:10.1371/journal.pone.0122513 (2015).

29 Wesslowski, J. *et al.* eGFP-tagged Wnt-3a enables functional analysis of Wnt trafficking and signaling and kinetic assessment of Wnt binding to full-length Frizzled. *J Biol Chem*, doi:10.1074/jbc.RA120.012892 (2020).

30 Proffitt, K. D. *et al.* Pharmacological inhibition of the Wnt acyltransferase PORCN prevents growth of WNT-driven mammary cancer. *Cancer Res* **73**, 502-507, doi:10.1158/0008-5472.CAN-12-2258 (2013).

31 Eubelen, M. *et al.* A molecular mechanism for Wnt ligand-specific signaling. *Science* **361**, doi:10.1126/science.aat1178 (2018).

32 Avet, C. *et al.* Selectivity Landscape of 100 Therapeutically Relevant GPCR Profiled by an Effector Translocation-Based BRET Platform. *bioRxiv*, 2020.2004.2020.052027, doi:10.1101/2020.04.20.052027 (2020).

## Supplementary Figures

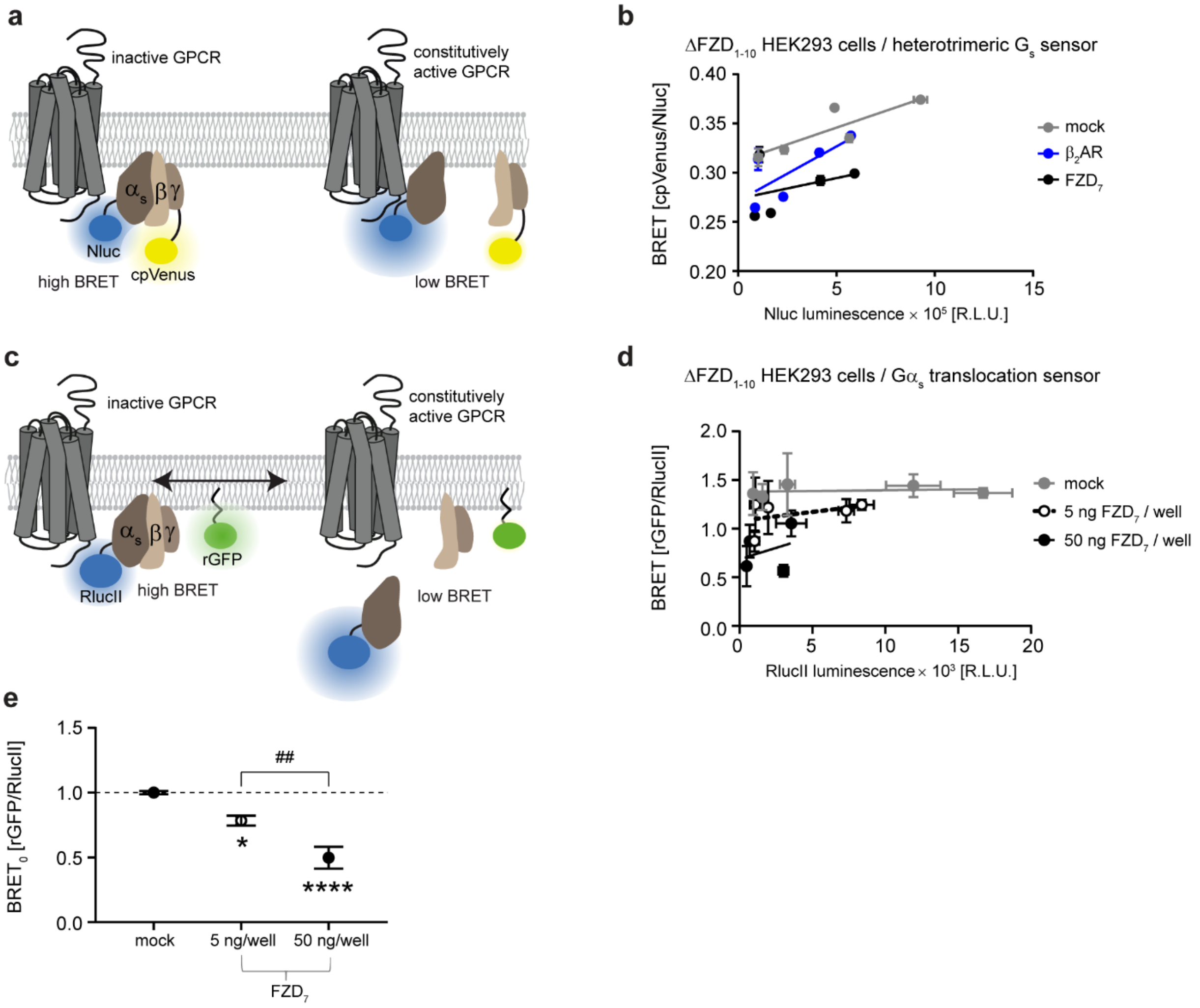


**Fig. S1 Constitutive activity of $FZD_7$ assessed by BRET-based $G_s$ biosensors.**
**a** Schematic of the BRET-based $G_s$ biosensor used to assess constitutive activity of GPCRs.
**b** Mean ± SD BRET values from five independent experiments (measured in quadruplicates) with the heterotrimeric $G_s$ biosensor carried out in $\Delta FZD_{1-10}$ HEK 293T cells were plotted against donor emission intensities to assess $BRET_0$ shown in **Fig. 1a**.
**c** Schematic of the BRET-based $G\alpha_s$ translocation assay.
**d-e** Independent BRET experiments carried out with the $G_s$ translocation sensor carried out in $\Delta FZD_{1-10}$ HEK 293T cells were plotted against donor emission in **d** and differences in constitutive activity were compared for two different transfection conditions (5 ng/well and 50 ng/well of $FZD_7$) in **e**. Data in **d** are represented as the mean ± SD and in **e** as the mean ± SEM (n=4-5, measured in quadruplicates). *$P < 0.05$; $^{\#\#}P < 0.01$; ****$P < 0.0001$ (one-way ANOVA followed by Sidak's multiple comparison).

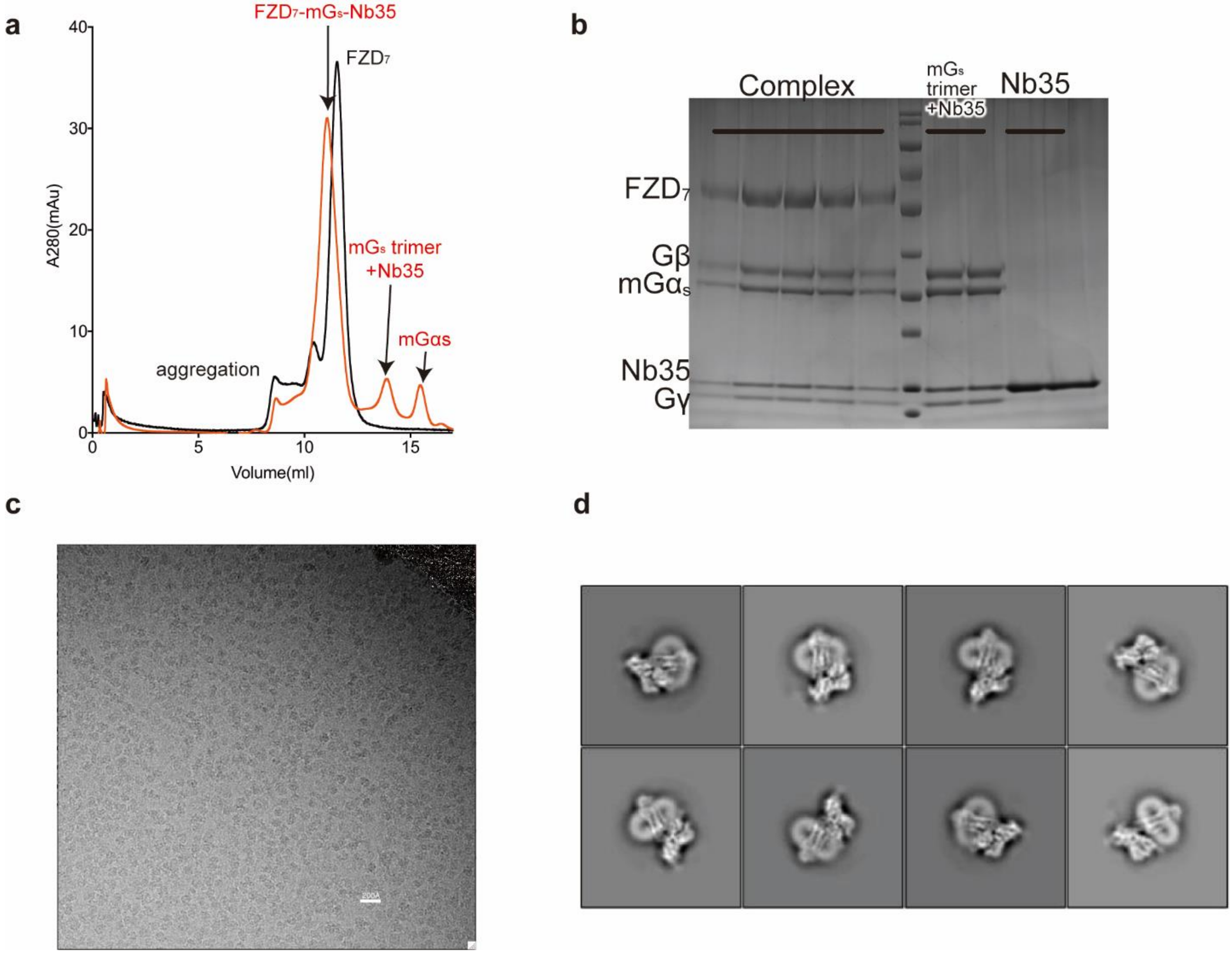


**Fig. S2 Assembly of the FZD$_7$-mG$_s$-Nb35 complex.**

**a** Superdex 200 size-exclusion chromatography elution profiles of the purified FZD$_7$-mG$_s$-Nb35 complex.

**b** SDS-PAGE/Coomassie stain analysis of the FZD$_7$-mG$_s$-Nb35 complex after size exclusion chromatography.

**c** Representative cryo-EM micrograph of the FZD$_7$-mG$_s$-Nb35 complex. Scale bar, 20 nm.

**d** Representative 2D class averages showing clear secondary features and different orientations of the particles. Box size, 270 Å.

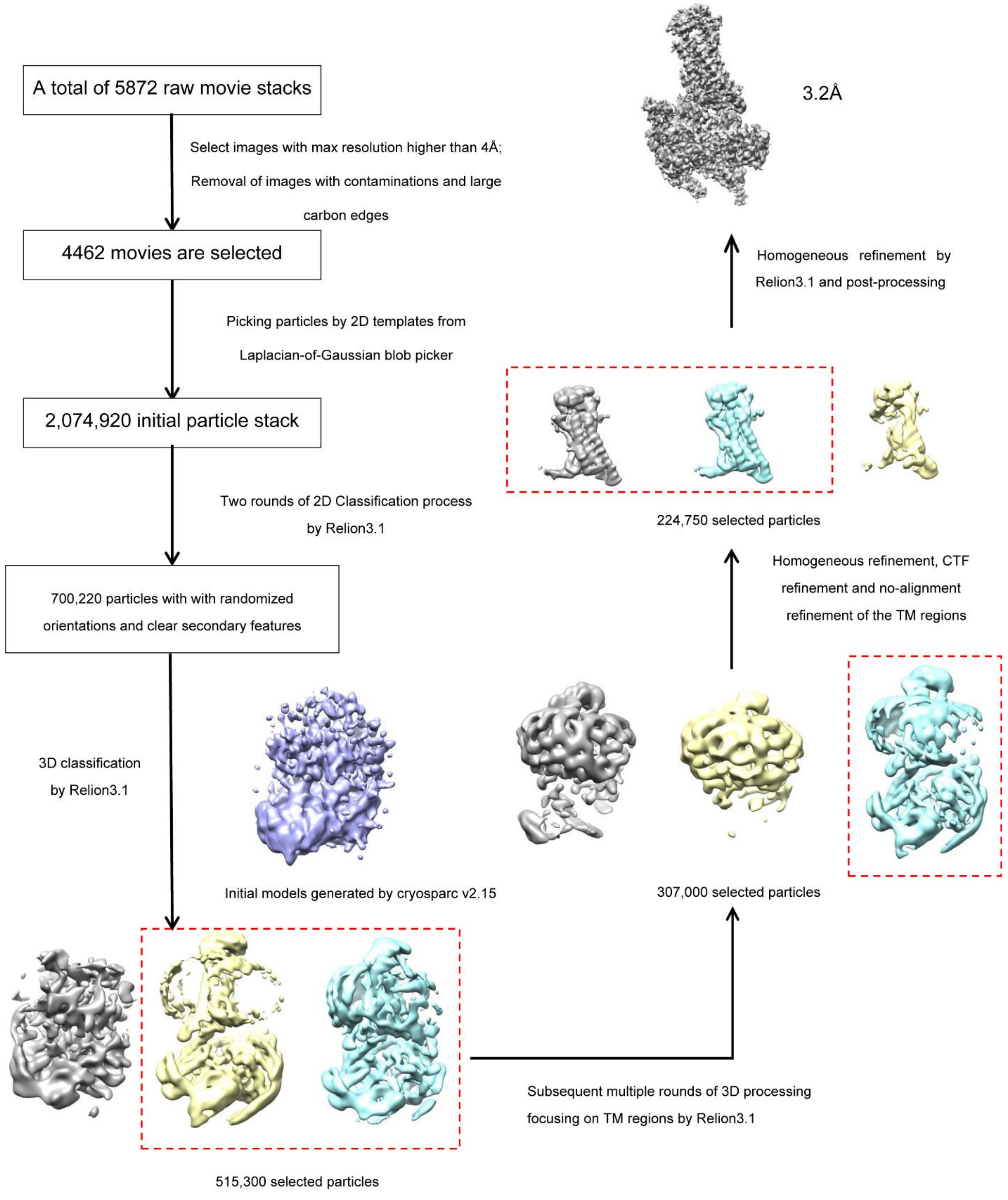


**Fig S3. Data processing of FZD$_7$-mG$_s$-Nb35 complex.**
Flow chart of cryo-EM data processing of the FZD$_7$-mG$_s$-Nb35 complex with the full map. The initial model was generated by cryoSPARC v2.15. The 2D and 3D classification and post-processing were processed by Relion3.1.

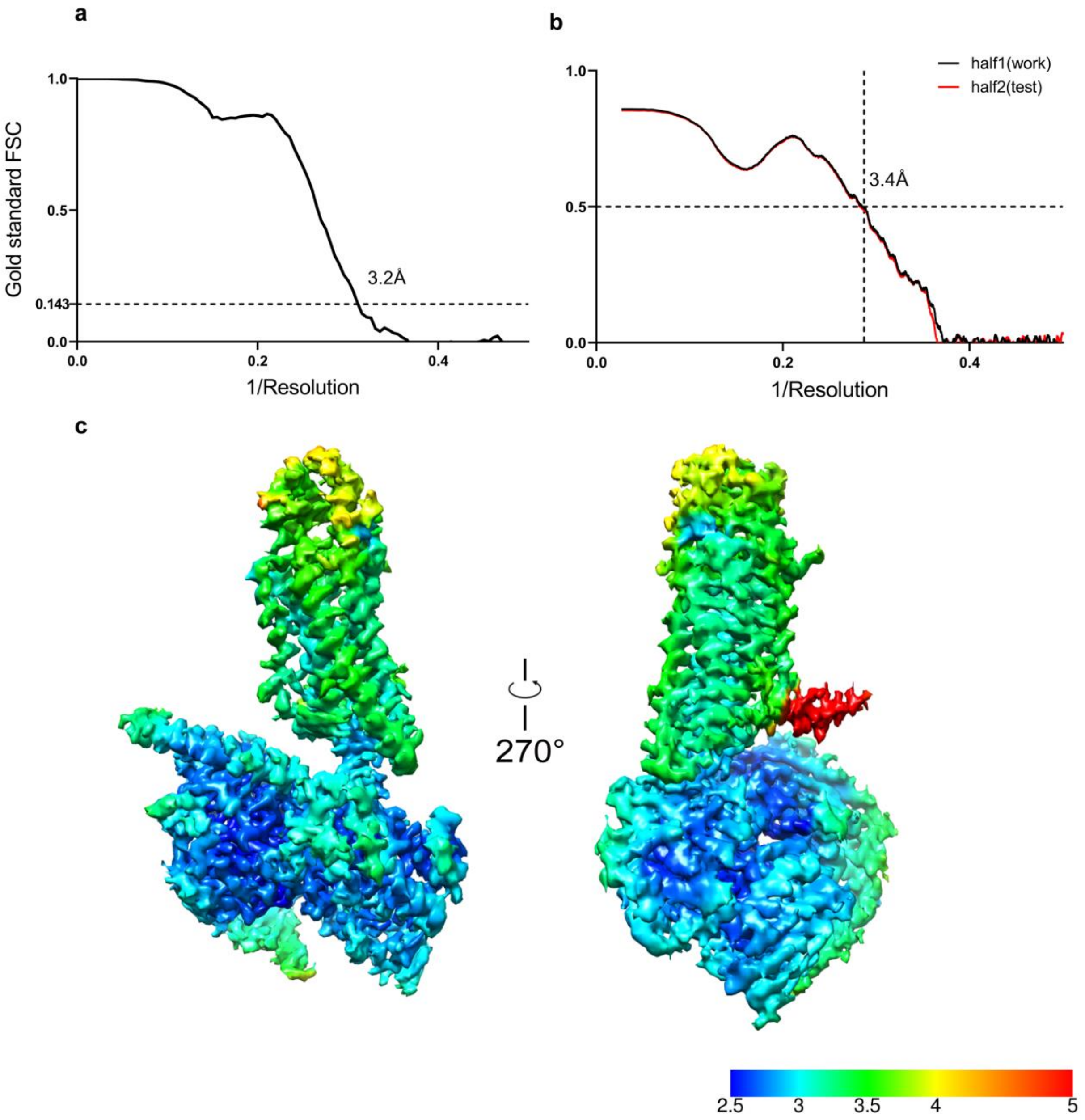


**Fig S4. Assessment of model quality.**

**a** Gold standard FSC curve of the $FZD_7$-$mG_s$-Nb35 complex.

**b** The FSC curves calculated between the refined structure and the half map used for model building (black, work) and the other half map (red, test).

**c** Density map of the side view of the structure, colored according to the local-resolution estimation.

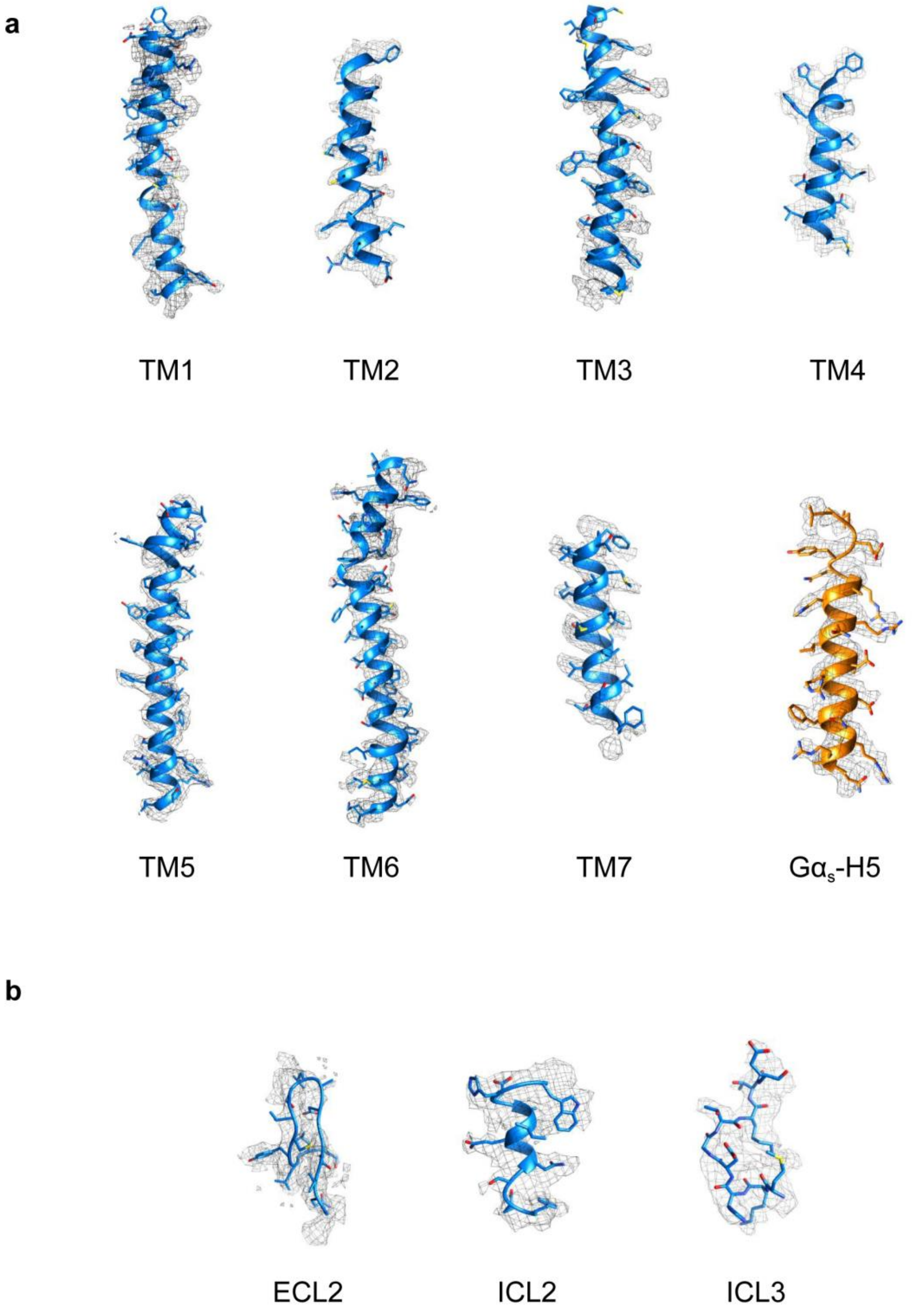


**Fig S5. Cryo-EM map of structural elements in the $FZD_7$-$mG_s$-Nb35 complex.**
**a** The segmented electron density maps of the transmembrane helices of $FZD_7$ and the α5-helix of the $mG\alpha_s$ superimposed with the models in respective regions.
**b** The ECL2, ICL2 and ICL3 of $FZD_7$.

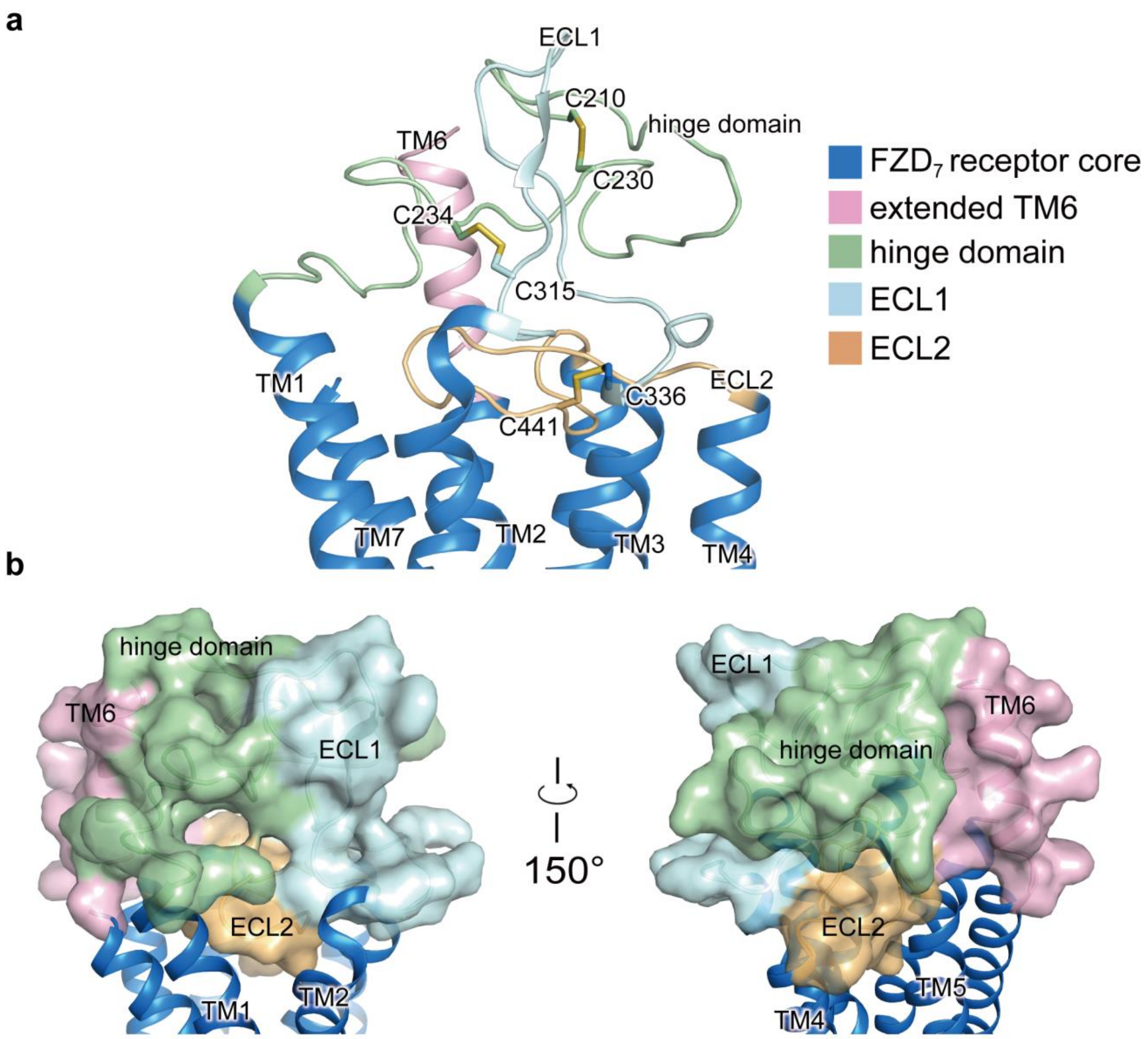


**Fig S6. The organization of the extracellular region in $FZD_7$ including hinge domain, ECL1, ECL2 and TM6 in the $FZD_7$-$mG_s$ structure.**

**a** C210-C230 (hinge domain), C234 (hinge domain)-C315 (ECL1) and C336 (ECL1)-C441 (ECL2) disulfide bonds are shown as sticks.

**b** $FZD_7$ receptor core: blue; hinge domain: green; ECL1: light blue; ECL2: orange; extended TM6: pink.

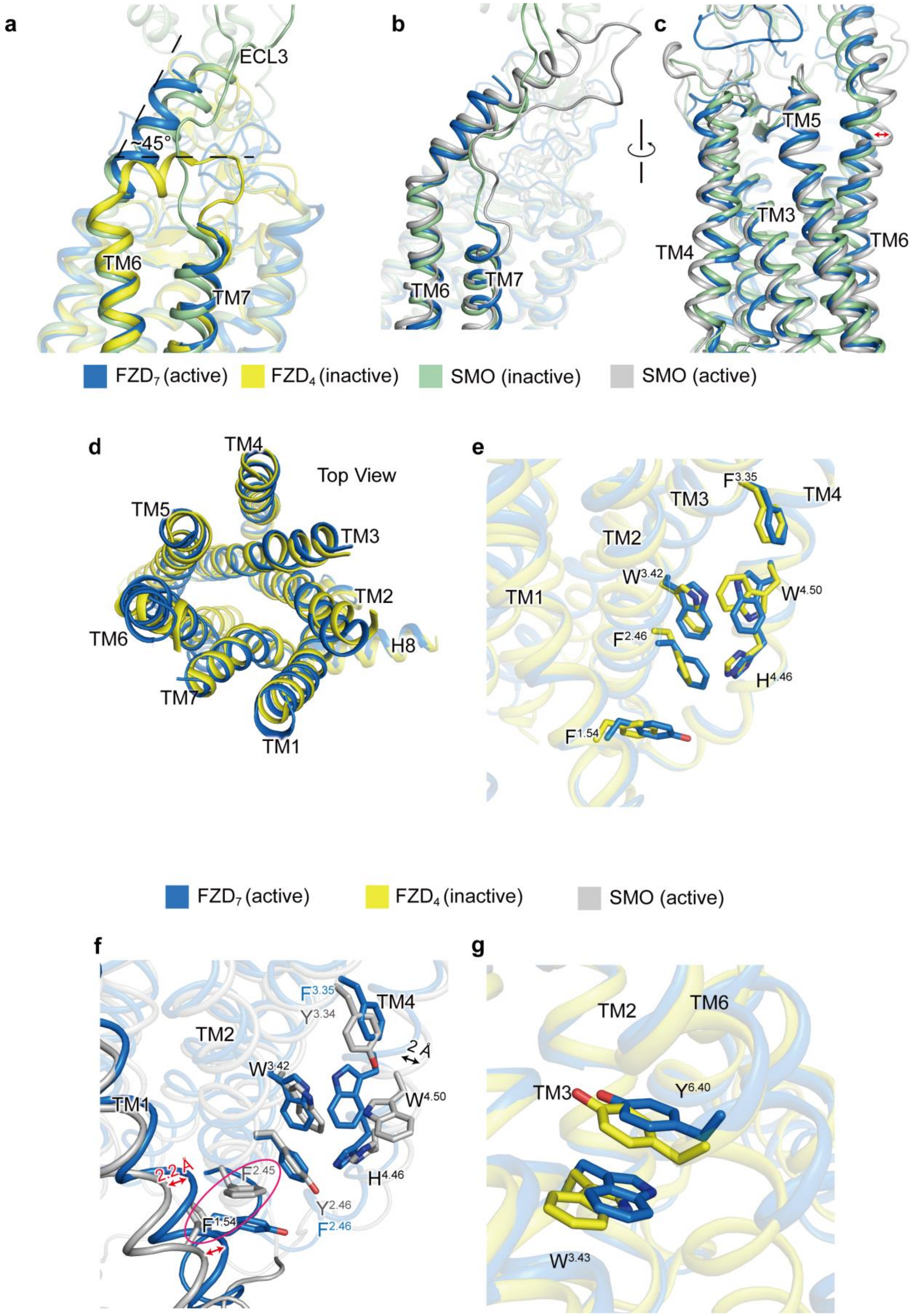

a
b
c
ECL3
~45°
TM6
TM7
TM6
TM7
TM5
TM3
TM4
TM6
FZD7 (active)
FZD4 (inactive)
SMO (inactive)
SMO (active)
d
e
TM4
Top View
TM5
TM3
TM2
TM6
H8
TM7
TM1
F3.35
TM3
TM4
TM2
W3.42
W4.50
TM1
F2.46
H4.46
F1.54
FZD7 (active)
FZD4 (inactive)
SMO (active)
f
g
F3.35
TM4
Y3.34
TM2
2 Å
W3.42
W4.50
TM1
2.2 Å
F2.45
H4.46
Y2.46
F1.54
F2.46
TM2
TM6
TM3
Y6.40
W3.43

**Fig S7. Comparison of $FZD_7$ with other class F GPCR structures.**
**a** Comparison of active $FZD_7$ structure (blue) with inactive $FZD_4$ structure (PDB: 6BD4, yellow) and inactive SMO structure (PDB: 5V57, green) with focus on TM6, TM7 and ECL3.
**b-c** Comparison of active $FZD_7$ structure (blue) with inactive SMO structure (PDB: 5V57, green) and active SMO structure coupled to $G_i$ (PDB: 6OT0, gray) with focus on TM6, TM7 and ECL3 in **b** and upper portion of TM2-TM6 bundle in **c**. Red arrows indicate the shift of TM6 between $FZD_7$ and SMO.
**d** Superposition of $FZD_7$ (blue) and $FZD_4$ (PDB: 6BD4, yellow) structures, viewed from the extracellular side (top view).
**e** $F^{1.54}$-$F^{2.46}$-$F^{3.35}$-$W^{3.42}$-$H^{4.46}$-$W^{4.50}$ π-cation network in $FZD_7$ and $FZD_4$.
**f** The π-π network in $FZD_7$ ($F^{1.54}$-$F^{2.46}$-$F^{3.35}$-$W^{3.42}$-$H^{4.46}$-$W^{4.50}$) and active SMO ($F^{1.54}$-$F^{2.45}$-$Y^{2.46}$, $Y^{3.34}$-$W^{3.42}$-$H^{4.46}$-$W^{4.50}$) structures. Red arrows indicate the outward movement of the TM bundle in active SMO structure compared to $FZD_7$.
**g** Comparison of $W^{3.43}$-$Y^{6.40}$ π-π interactions in $FZD_7$ (blue) and $FZD_4$ (yellow).

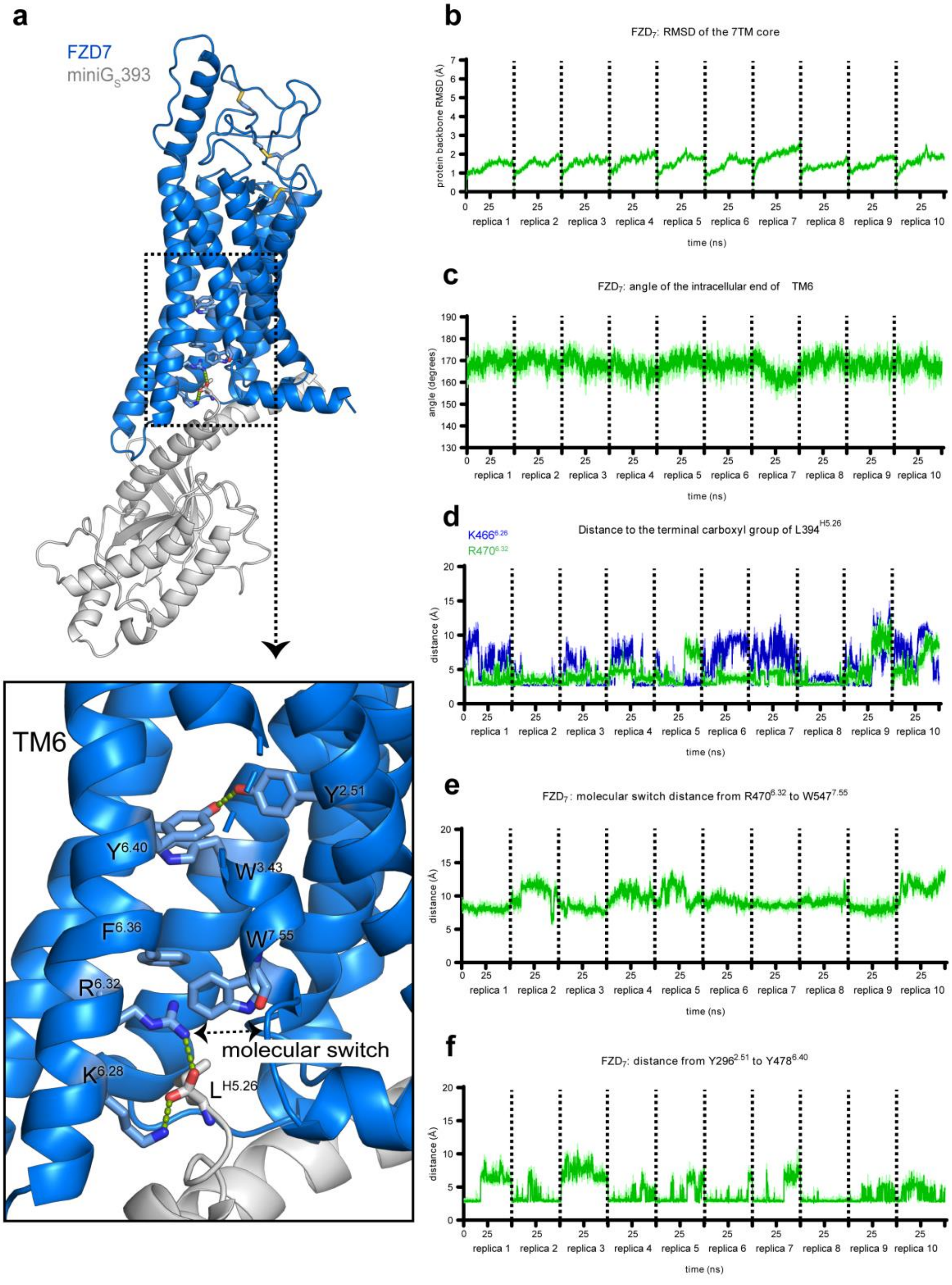

a
FZD7
miniGs393
TM6
Y2.51
Y6.40
W3.43
F6.36
W7.55
R6.32
molecular switch
K6.28
LH5.26
b
FZD7: RMSD of the 7TM core
protein backbone RMSD (Å)
replica 1 replica 2 replica 3 replica 4 replica 5 replica 6 replica 7 replica 8 replica 9 replica 10
time (ns)
c
FZD7: angle of the intracellular end of TM6
angle (degrees)
time (ns)
d
K466 6.26
R470 6.32
Distance to the terminal carboxyl group of L394 H5.26
distance (Å)
time (ns)
e
FZD7: molecular switch distance from R470 6.32 to W547 7.55
distance (Å)
time (ns)
f
FZD7: distance from Y296 2.51 to Y478 6.40
distance (Å)
time (ns)

**Fig S8. Molecular dynamics simulations of $FZD_7$.**

**a** The active $FZD_7$ structure (blue) in complex with $mG_s393$ model (white) after 50 ns of equilibration MD (i.e. at simulation t = 0). The lower part of the panel presents a zoom-in to the extended molecular switch. The production MD was run for 50 ns in 10 independent replicas. Snapshots of t=0 and the ten replica t=50 ns are provided as supplementary data files.

**b** The protein backbone RMSD of $FZD_7$ (7TM helices, helix 8 and ICLs 1-3) stabilizes within a window of 1 Å in all simulation trajectories, underlining the robustness of the $FZD_7$-$mG_s$ structure. The linker and ECLs are excluded from the analysis to avoid misinterpretations originating from the flexibility of the original structure at these areas (manifested by the unresolved ICL3 and CRD).

**c** The angle of the intracellular part of TM6 measured between the backbone nitrogen atoms of $V485^{6.47}$, $P481^{6.43}$ and $E462^{6.24}$ (corresponding to the end of TM6).

**d** The distance between the C-terminal carboxyl group of $L394^{H5.26}$ of the α5 helix of $mG_s393$ and $K466^{6.28}$ (blue) or $R470^{6.32}$ (green) of $FZD_7$.

**e** The distance between $R470^{6.32}$ and the backbone oxygen atom of $W547^{7.55}$ in the molecular switch.

**f** The distance between $Y296^{2.51}$ and $Y478^{6.40}$.

In addition to the local opening of the lower portions of TM6 and TM7 by disruption of the molecular switch ($R^{6.32}$ and $W^{7.55}$), all of the active SMO structures (PDB IDs: 6OT0, 6O3C and 6XBL, 6XBJ, 6XBK and 6XBM) exhibit a network of aromatic interactions that is established by the parallel outward movement of TM6. In SMO, this network is composed of $W^{7.55}$, $F^{6.36}$ and $F^{3.43}$ and it is likely to stabilize the active receptor conformation as it is not intact in any of the inactive SMO structures (PDB IDs: 4QIM, 4O9R, 4N4W, 4JKV and 5L7I). In support of an intramolecular network that stabilizes the active conformation, similar but extended aromatic interactions are formed by $W^{7.55}$, $F^{6.36}$, $W^{3.43}$ and $Y^{6.40}$ in the $FZD_7$-$mG_s$ complex. In SMO, position 6.40 is an alanine, which is not involved in these interactions due to the lack of π electrons. Quite unexpectedly, $Y478^{6.40}$ of FZD7 could form a hydrogen bond with $Y296^{2.51}$ (distance between the hydroxyl-oxygen atoms is ca. 4 Å); this contact is present in both currently available inactive FZD structures ($FZD_4$ and $FZD_5$; PDB IDs 6BD4 and 6WW2, respectively) and could stabilize the inactive receptor conformation. In MD simulations, however, the $Y478^{6.40}$-$Y296^{2.51}$ interaction is more transient and occurs only in approximately 50% of the MD frames. It is possible that a presence of an agonist ligand could promote further the opening of this intramolecular contact.

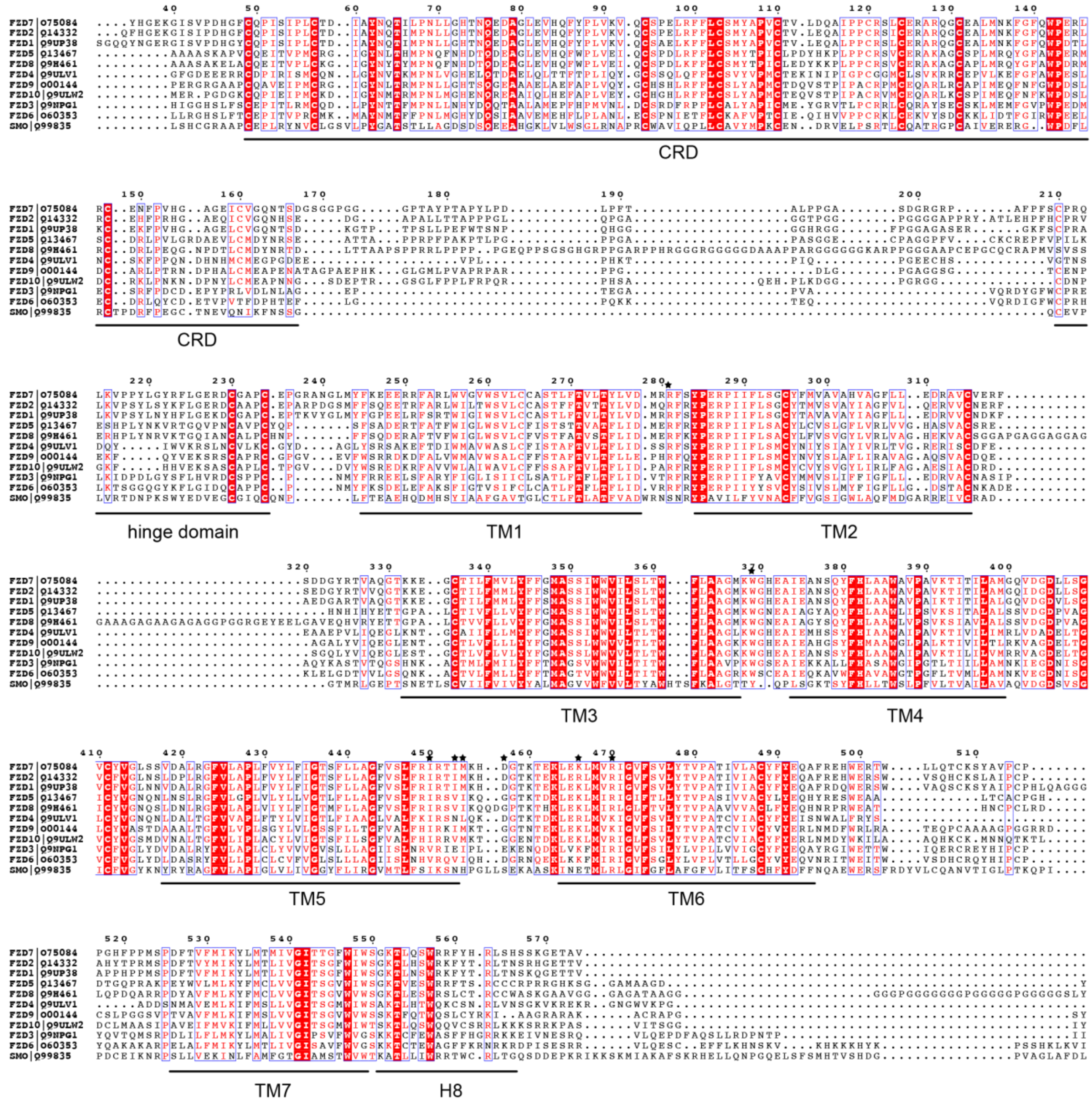


**Fig S9. Sequence alignment between $FZD_7$ and other class F members including SMO.**

Colors represent the similarity of residues: red background: identical; red text: strongly similar. The alignment was generated using MAFFT (https://www.ebi.ac.uk/Tools/ms/mafft/) and the graphic was prepared on the ESPript 3.0 server (https://espript.ibcp.fr/ESPript/cgi-bin/ESPript.cgi). Residues ($R281^{ICL1}$, $W370^{ICL2}$, $I450^{5.72}$, $I453^{5.75}$, $M454^{5.76}$, $D457^{ICL3}$, $K466^{6.28}$ and $R470^{6.32}$) interacting with $mG\alpha_s$ are labeled with a black star.

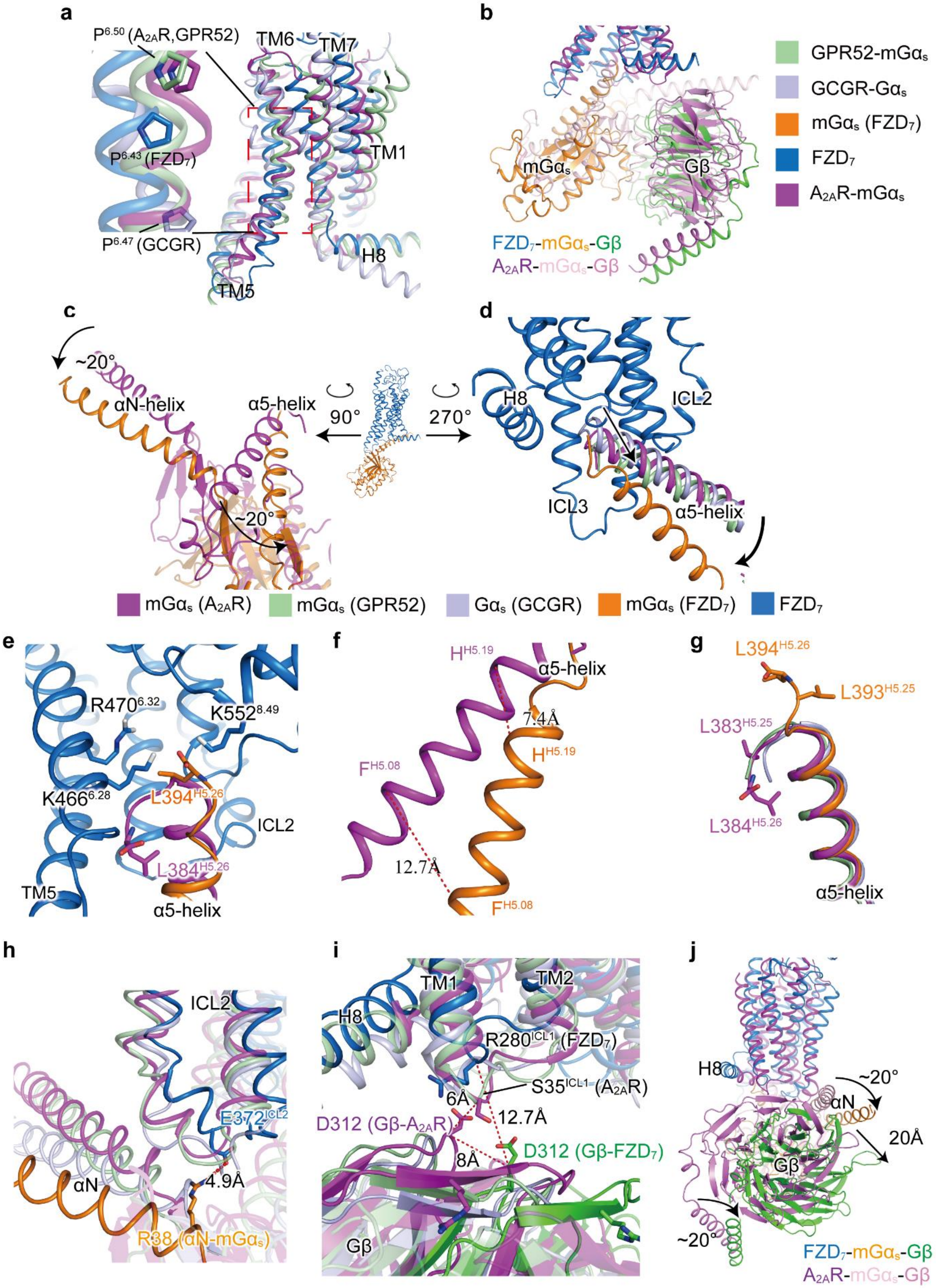

a
P6.50 (A2AR,GPR52)
TM6
TM7
P6.43 (FZD7)
TM1
P6.47 (GCGR)
H8
TM5
b
GPR52-mGαs
GCGR-Gαs
mGαs (FZD7)
FZD7
A2AR-mGαs
mGαs
Gβ
FZD7-mGαs-Gβ
A2AR-mGαs-Gβ
c
~20°
αN-helix
α5-helix
90°
~20°
d
270°
H8
ICL2
ICL3
α5-helix
mGαs (A2AR)
mGαs (GPR52)
Gαs (GCGR)
mGαs (FZD7)
FZD7
e
R470 6.32
K552 8.49
K466 6.28
L394 H5.26
ICL2
L384 H5.26
TM5
α5-helix
f
H H5.19
α5-helix
7.4Å
H H5.19
F H5.08
12.7Å
F H5.08
g
L394 H5.26
L393 H5.25
L383 H5.25
L384 H5.26
α5-helix
h
ICL2
E372 ICL2
αN
4.9Å
R38 (αN-mGαs)
i
TM1
TM2
H8
R280 ICL1 (FZD7)
S35 ICL1 (A2AR)
6Å
12.7Å
D312 (Gβ-A2AR)
8Å
D312 (Gβ-FZD7)
Gβ
j
H8
~20°
αN
20Å
Gβ
~20°
FZD7-mGαs-Gβ
A2AR-mGαs-Gβ

**Fig S10. Comparison of $FZD_7$-$mG_s$-Nb35 complex with $A_{2A}R$-$mG_s$, GPR52-$mG_s$ and GCGR-$G_s$ structures.**

GPCR-G protein complex structures for: $FZD_7$-mG$\alpha_s$-Gβ (blue-orange-green), $A_{2A}R$-$mG_s$ (magenta; 6GDG), GPR52-$mG_s$ (green; 6LI3) and GCGR-$G_s$ (gray; 6WPW).
**a** Superposition of receptor structures in the four complexes.
**b** Superposition of $FZD_7$ with $A_{2A}R$ bound to mG$\alpha_s$ and Gβ (Gγ and Nb35 were omitted for clarity).
**c** Comparison of α5-helices and αN-helices between $FZD_7$-$mG_s$ and $A_{2A}R$-$mG_s$ complexes.
**d** Positions of α5-helices in the four GPCR-G protein complex structures relative to $FZD_7$.
**e** Interactions centered around the C-terminal tail of α5-helices in $FZD_7$-$mG_s$ and $A_{2A}R$-$mG_s$ complexes. The N atom of $R470^{6.23}$, $R281^{ICL1}$ and $R466^{6.28}$ are colored gray.
**f** The clockwise rotation of the α5-helix in $FZD_7$-$mG_s$ from that in $A_{2A}R$-$mG_s$ and the downward shifting distances (measured at $H^{H5.19}$ or $F^{H5.08}$) in these two superimposed models.
**g** The uncoiled and straight-up conformation of the C-terminal tail of α5-helix in $FZD_7$-$mG_s$ aligned to the other three GPCR-G protein complexes.
**h** Comparison of interactions between ICL2 of each GPCR and the αN of G$\alpha_s$/mG$\alpha_s$ in four structures. Distance between $E372^{ICL2}$ of $FZD_7$ and R38 of mG$\alpha_s$ in $FZD_7$-$mG_s$-Nb35 complex is labeled.
**i** Comparison of the distance between Gβ (measured on D312, the closest point to the receptor) and GPCRs in $FZD_7$-$mG_s$ with that in $A_{2A}R$-$mG_s$ structure. The distance between $R280^{ICL1}$ and D312 (Gβ) in $FZD_7$-$mG_s$ is 12.7Å whereas the distance between $S35^{ICL1}$ and D312 (Gβ) in $A_{2A}R$-$mG_s$ is 6Å. D312 on Gβ in the two structure is shifted by 8Å.
**j** Rotation of mG$\alpha_s$ and Gβ in $FZD_7$-$mG_s$ and $A_{2A}R$-$mG_s$ structures.

$FZD_7$-$mG_s$ shows some distinct features from other representative GPCR-$G_s$ complexes, including the class A adenosine $A_{2A}$ receptor ($A_{2A}R$)-$mG_s$ (PDB: 6GDG), the orphan receptor GPR52-$mG_s$ (PDB: 6LI3) and the class B glucagon receptor GCGR-$G_s$ (PDB: 6WPW), particularly at the $FZD_7$-$mG_s$ interface. The whole $mG_s$-trimer and Nb35 module rotates by more than 20 °when comparing the $FZD_7$-$mG_s$ to the $A_{2A}R$-$mG_s$ complex (measured on the α5-helix when the two receptors are aligned), and shifts downward by about 7 Å (measured on $H^{5.19}$ in α5-helix in the two complexes). The twist and downward shifting of the whole module is promoted by the interaction mode of the α5-helix of mG$\alpha_s$ with $FZD_7$ and results in a greater distance between the ICL1/ICL2/H8 and the Gα-Gβ surface in $FZD_7$-$mG_s$ when compared to $A_{2A}R$-$mG_s$ and other receptor-$G_s$ complexes. The partially uncoiled and elongated C-terminus of the α5-helix points upward and interacts with multiple $FZD_7$ residues. Here, $FZD_7$ $K466^{6.28}$ and $R470^{6.32}$ and the last two leucine residues of the α5-helix play a central role in stabilizing the interaction network. Also, while there are multiple interfaces from heterotrimeric $G_s$, including the αN helix of G$\alpha_s$ and Gβ, in other GPCR-$G_s$ complexes, the interface between $FZD_7$ and $mG_s$ is dominated by the α5-helix-mediated interactions. More specifically, we only observed a weak contact between R38 on the αN-helix of mG$\alpha_s$ and E372 on ICL2 of $FZD_7$.

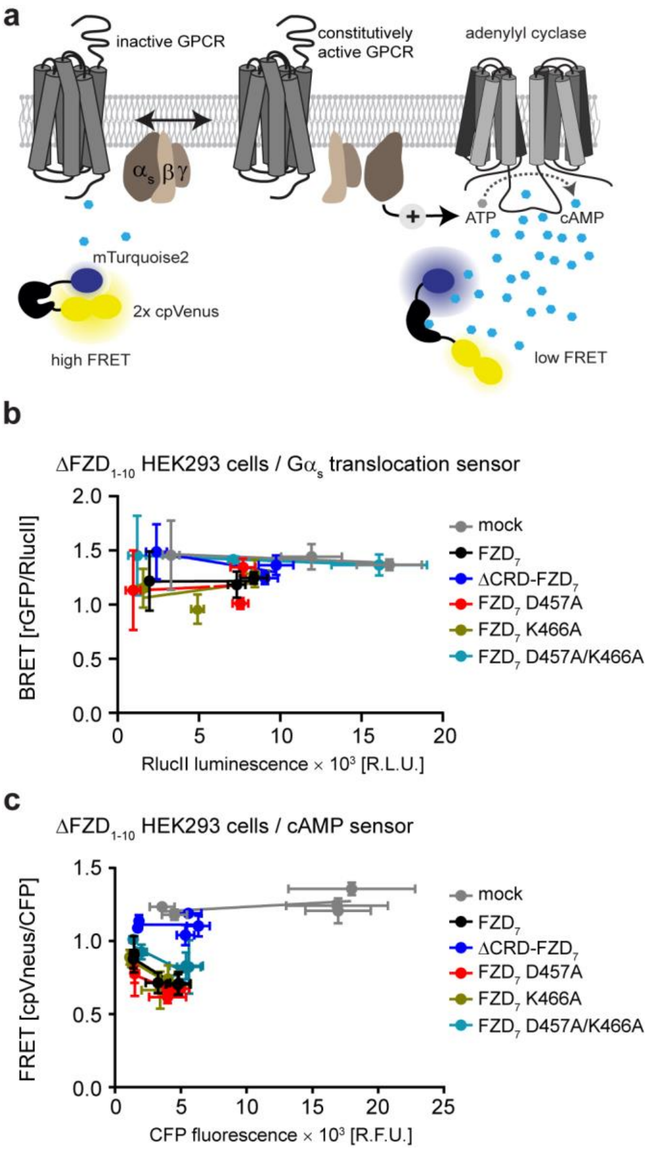


**Fig S11. RET data from biosensor experiments plotted against donor emission.**

**a** Schematic of the FRET-based cAMP accumulation assay.
**b-c** Independent biosensor experiments with the G$\alpha_s$ translocation sensor in **b**, and the cAMP sensor in **c** carried out in $\Delta$FZD$_{1\text{-}10}$ HEK 293T cells. Data are represented as the mean ± SD (n=4-5, measured in quadruplicates).

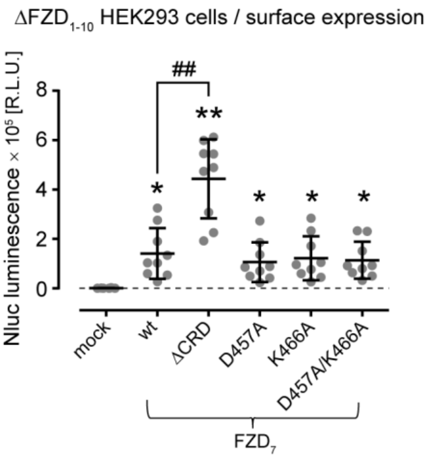


**Fig S12. Surface expression of HiBiT-FZD$_7$ and associated mutants used in biosensor experiments.**

HiBiT-tagged constructs were expressed in ΔFZD$_{1-10}$ HEK 293T cells and cell surface expression was quantified using the Nano-Glo® HiBiT Extracellular Detection System. Data are represented as the mean ± SEM (n=6-9, measured in triplicates). ns non-significant; ****P < 0.0001 (one-way ANOVA).

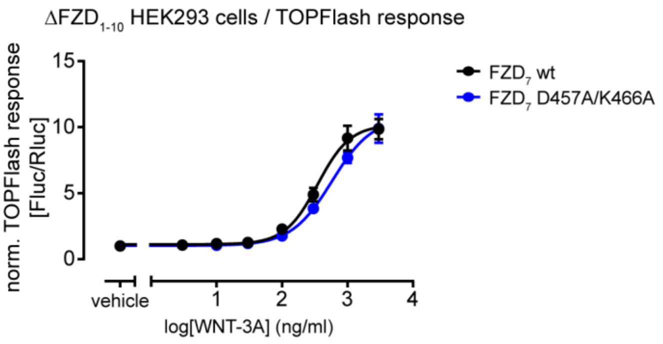


**Fig. S13 WNT-induced β-catenin signaling mediated by FZD$_7$ wildtype or the double mutant D457A/K466A.**

Normalized TOPFlash reporter gene response mediated by FZD$_7$ wildtype or the double mutant D457A/K466A upon stimulation with increasing concentrations of recombinant WNT-3A. Data are represented as the mean ± SEM (n=6, measured in duplicates).

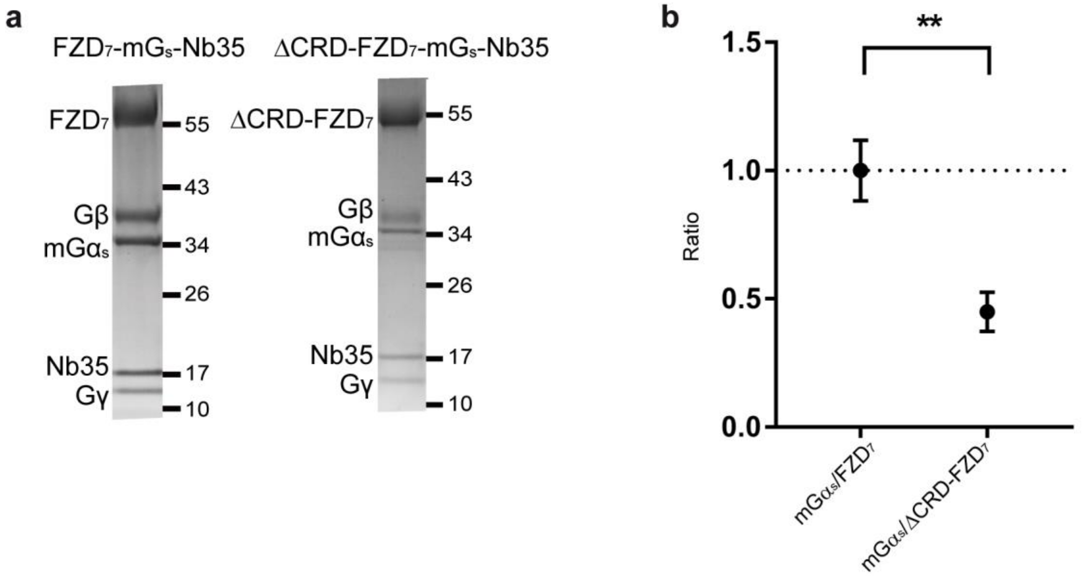


**Fig S14. Biochemical assessment of $FZD_7$-m$G_s$ and ΔCRD-$FZD_7$-m$G_s$ complex formation.**
**a** SDS-PAGE analysis of $FZD_7$-m$G_s$-Nb35 complex and ΔCRD-$FZD_7$-m$G_s$-Nb35 complex after size-exclusion chromatography.
**b** Quantitative analysis of the complex formation. Data are represented as the mean ± s.e.m. of the ratio of band intensity obtained from SDS-PAGE for the m$G\alpha_s$/$FZD_7$ pair (from three independent experiments) and the m$G\alpha_s$/ΔCRD-$FZD_7$ pair (from four independent experiments). The ratio of m$G\alpha_s$/$FZD_7$ was set to "1". $^{**}P < 0.01$ (Student's t test).

**Supplementary Table**

**Table S1. Cryo-EM data collection, refinement and validation statistics**

| | $FZD_7$-$mG_s$-Nb35 complex |
|---|---|
| **Data collection and processing** | |
| Magnification | 130,000 |
| Voltage (kV) | 300 |
| Electron exposure ($e^-/Å^2$) | 60 |
| Defocus range (um) | -0.7 ~ -2.2 |
| Pixel size (Å) | 1.04 |
| Symmetry imposed | C1 |
| Initial particle images (no.) | 2,074,920 |
| Final particle images (no.) | 224,750 |
| Map resolution (Å) | 3.2 |
| FSC threshold 0.143 | |
| Map resolution range (Å) | 2.4~7.5 |
| | |
| **Refinement** | |
| Initial model used (PDB code) | 5V57, 7D3S, 3CIK, 6PCV and 6GDG |
| Map sharpening B factor ($Å^2$) | -100 |
| Model composition | |
| Non-hydrogen atoms | 8670 |
| Protein Residues | 1094 |
| Ligands | 0 |
| B factor ($Å^2$) | |
| Protein | 72.15 |
| Ligand | n/a |
| R.m.s. deviations | |
| Bond lengths (Å) | 0.004 |
| Bond angles (°) | 0.756 |
| Validation | |
| MolProbity score | 1.73 |
| Clashscore | 7.34 |
| Poor rotamers (%) | 0.00 |
| Ramachandran plot | |
| Favored (%) | 95.28 |
| Allowed (%) | 4.72 |
| Disallowed (%) | 0.00 |
